# Photoelectrochemistry and Drift–Diffusion Simulations in a Polythiophene Film Interfaced with an Electrolyte

**DOI:** 10.1021/acsami.1c10158

**Published:** 2021-07-26

**Authors:** Greta Chiaravalli, Giovanni Manfredi, Riccardo Sacco, Guglielmo Lanzani

**Affiliations:** † Center for Nano Science and Technology, Istituto Italiano di Tecnologia, 20133 Milan, Italy; ‡ Department of Physics, Politecnico di Milano, 20133 Milan, Italy; § Department of Mathematics, Politecnico di Milano, 20133 Milan, Italy

**Keywords:** bioelectronics, polythiophenes, photoelectrochemistry, drift−diffusion models, solid−liquid interface

## Abstract

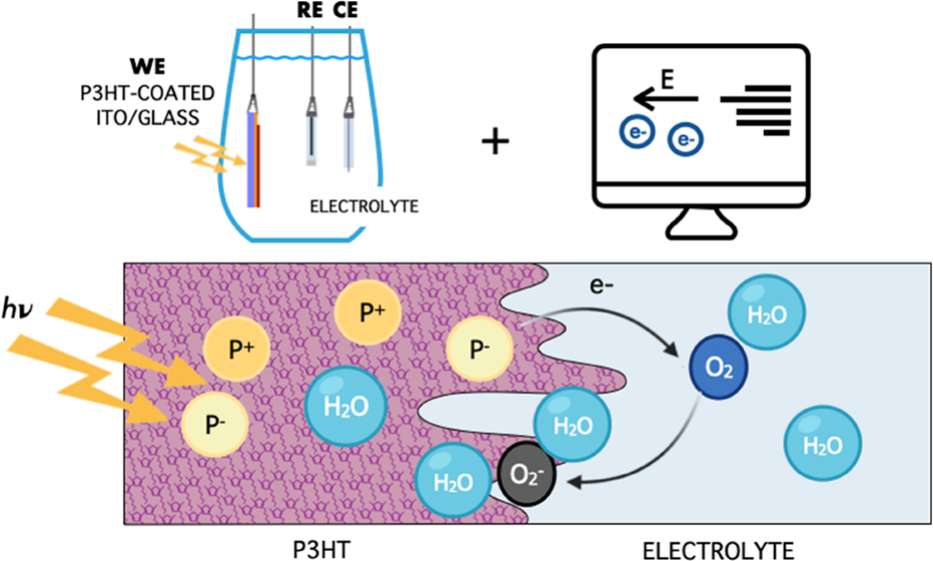

Although the efficiency of organic polymer-based retinal devices has been proved, the interpretation of the working mechanisms that grant photostimulation at the polymer/neuron interface is still a matter of debate. To contribute solving this issue, we focus here on the characterization of the interface between poly(3-hexyltiophene) films and water by the combined use of electrochemistry and mathematical modeling. Simulations well reproduce the buildup of photovoltage (zero current condition) upon illumination of the working electrode made by a polymer film deposited onto an indium tin oxide (ITO) substrate. Due to the essential unipolar transport in the photoexcited film, diffusion leads to a space charge separation that is responsible for the initial photovoltage. Later, electron transfer reactions toward oxygen in the electrolyte extract negative charge from the polymer. In spite of the simple model studied, all of these considerations shed light on the possible coupling mechanisms between the polymeric device and the living cell, supporting the hypothesis of pseudocapacitive coupling.

## Introduction

Light is a noninvasive, high-resolution regulatory tool that may find important applications in healing neurodegenerative disorders of the central nervous system or in in vitro studies. Because of neuron transparency, specific actions are needed to achieve neuronal light stimulation. The general approach consists in the realization of a smart abiotic/biotic interface able to transduce a light stimulus into a bioelectrical^1^ or biochemical^2,3^ signal. Another approach is to use infrared light to perturb the cell membrane dynamical equilibrium.^4^ New strategies are based on organic materials and configurations where the external source of energy is light, including the use of nanotechnology.^5−12^ Particular interest aroused from the use of a photovoltaic polymer layer to stimulate retinal neurons in vivo.^10^ In the proposed device, the poly(3-hexyltiophene) (P3HT) layer establishes a tight contact with the neuron membrane, possibly mediated by a thin solution cleft filled by proteins. Such an interface is virtually seamless because it is the contact between two carbon-based soft materials which support both ionic and electronic transport. This approach does not require gene therapy and does not directly interfere with ionic mechanisms; instead, it acts upon the passive properties of the membrane. Although the efficiency of P3HT-based retinal devices has been proven, there is no conclusive interpretation on the working mechanisms that grant photostimulation^9^ and which are supposedly related to electrostatic effects arousing at the interface of the material. To clear out some of the processes that occur in these devices upon illumination, in the present work we study the fundamental interface structure, namely, polymer/electrolyte interface. Despite being an oversimplified model of the in vivo interface, it provides the ground for capturing essential ingredients in the physics of the interface that can be further declined in following up models. In addition, we assess the role of oxygen whose concentration in the retina is a matter of debate and can be a crucial parameter in the in vivo experiments.^13^

**Figure 1:**
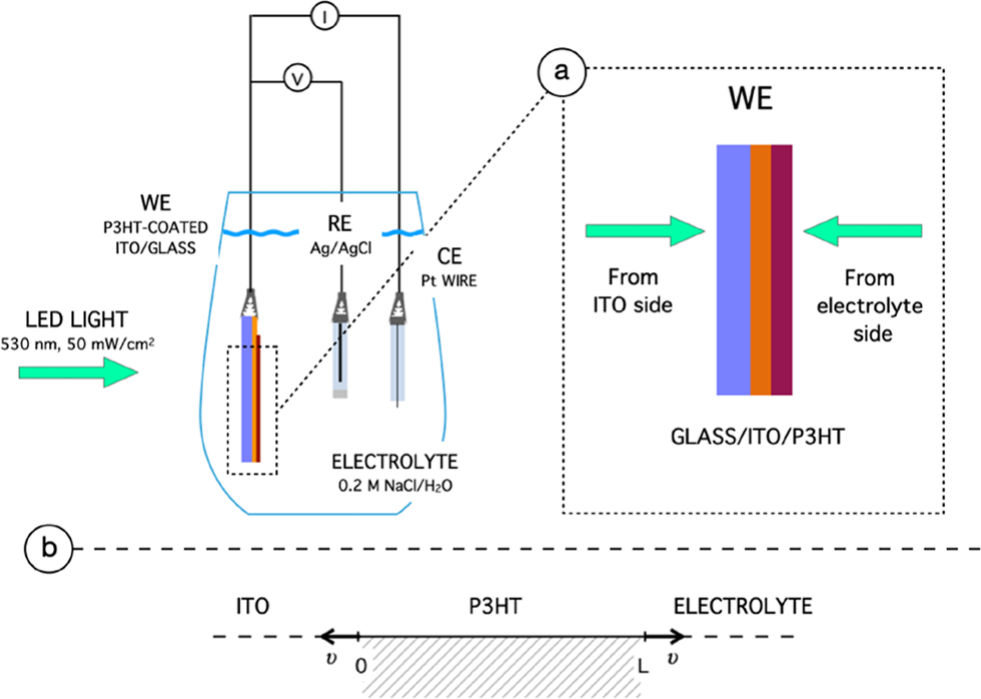
Experimental setup and sample. The figure shows the scheme of a three-electrode electrochemical setup: the working electrode (WE) corresponds to our sample, the reference electrode (RE) to an electrode of Ag/AgCl sat., and the counter electrode (CE) to a platinum wire. In our experiment, the total current circulating is set equal to 0 by the amperometer I and the change in the voltage due to illumination between the WE and RE is measured by the voltmeter V. Zoom (a) shows the composite layered structure of the WE, made of glass, indium tin oxide (ITO), and a coating of P3HT of various thicknesses and the possible directions of illumination, from the ITO side or from the electrolyte side. Panel (b) shows the one-dimensional (1D) domain used to model the P3HT–electrolyte interface.

We combine mathematical simulations as well as experiments, mainly based on electrochemical techniques (Figure 1), to study photovoltage (PV) generated by an illuminated P3HT working electrode (WE) in an electrochemical cell. The mathematical model is based on the drift–diffusion equations.^14^ Successful examples of similar approaches can be found in the study of dye-sensitized solar cells,^15−17^ where indeed a solid–liquid interface is at the core of the device. Whether electrochemical studies are usually performed to characterize the response of the system to certain voltage/current application or to certain electrolytic environment, our goal is instead to obtain a quantitative description of the fundamental mechanisms related to the photoactivation of P3HT.

Combining the electrochemical experiment and drift–diffusion simulations, we obtain a picture of the charged photoexcitation dynamics in P3HT, as a function of time, oxygenation, and illumination. Our results suggest a coupling mechanism between the abiotic/biotic interface and pave the way for further modeling developments of such an interface.

## Methods

### Theoretical Model

The theoretical work is based on the use of a time-dependent version of the drift–diffusion model to describe the transport mechanisms of P3HT in a 1D domain where the ITO side is at *x* = 0 and the P3HT–electrolyte interface is at *x* = *L* (Figure 1b). The use of the drift–diffusion equations to effectively model the organic semiconductor is well accepted.^18,19^ The mathematical model consists of two continuity equations accounting for the drift and diffusion of photogenerated holes and electrons and a Poisson equation for the electric field and potential. The backward Euler difference method is used for time advancement. Then, at each discrete time level, the nonlinear partial differential equation system is iteratively solved by means of a novel variant of Gummel’s decoupled algorithm customarily used in the semiconductor device simulation.^14,20^ Finally, piecewise linear continuous finite elements are used to discretize the linearized equations. To ensure strict positivity of the computed carrier densities, the Scharfetter–Gummel stabilization is introduced in the continuity equations.^21,22^ The computational algorithm is inhouse coded and has been implemented using the MatLab scientific environment.

When light is off, we assume an electroneutral initial concentration of free carriers (*n*_i_ = 10^12^ m^–3^) with zero electric field across the bulk of the P3HT. Upon light absorption, neutral singlet states quickly decay away, while a small fraction of charge carriers survives giving rise to a steady-state population. Bulk charge carrier photogeneration has been documented in regioregular (RR) P3HT.^23^ Briefly RR-P3HT forms crystalline lamellae where wave function delocalization through π-stacking favors charge separation, similar to the special pair in photosynthesis. Bulk photogeneration of long-lived charge carriers has small quantum yield (see below) yet not negligible. On the contrary, we find that the electron transfer to ITO can be neglected.

The role of ITO, if any, is to provide a source of singlet quenching by image dipole energy transfer. The generation of charge carriers is described with the use of the Lambert–Beer law, while the recombination process is described with both the bimolecular annihilation process and the trap-mediated Shockley–Read–Hall model.^24^ The boundary conditions at *x* = 0 impose the experimental conditions of zero total current, namely, the sum of conduction and displacement component is set equal to zero.^25,26^ In our case, both the components are set to zero separately. Accordingly, the electric field is also set equal to 0 at this interface. The evaluation of the photovoltage is also performed at *x* = 0, according to PV = ψ(*x* = 0), ψ being the electric potential. We assume that photogenerated negative carriers reduce molecular oxygen at the interface between P3HT and the electrolyte.^27,28^ The exit of electrons from the bulk is described using the Marcus–Gerischer theory^29−32^
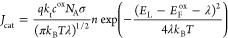
1where *q* is the elementary charge [C], *k*_t_ is the tunneling constant [m^4^ s^–1^], *c*^ox^ is the concentration of molecular oxygen at the interface [mol m^–3^], *N*_A_ is the Avogadro constant [mol^–1^], σ is the disorder parameter of P3HT [eV], *k*_B_ is the Boltzmann constant [J K^–1^], *T* is the room temperature [K], and λ is the width of the Gaussian distribution of molecular oxygen states [eV]. *E*_L_ is the energy of the lowest unoccupied molecular orbital (LUMO) of P3HT, *E*_F_^ox^ is the energy corresponding to the potential of the oxygen reduction reaction, and *n* is the electron number density at *x* = *L* [m^–3^]. By changing the value of *c*^ox^, we have been able to simulate different oxygenating conditions. To close the system of equations, at *x* = *L* the potential has been fixed equal to the experimental open circuit potential. The whole model is described in detail in Supporting Information Sections S1 and S2.

### Sample Preparation

Samples were produced by spin coating. ITO substrates (Ossila S281) were first cleaned using acetone, water, and isopropanol. After cleaning, samples were exposed to oxygen plasma for 1 min using a Plasma Etcher (Diener FEMTO Q, 0.12 mBar base pressure, 0.4 mBar O_2_ pressure, 100 W). A 30 mg mL^–1^ solution of P3HT in chlorobenzene was held at 50 °C and spin-coated over the ITO substrates at 800/1600/3200 rpm to obtain thick, intermediate, and thin samples, respectively. The rotation was held for 60 s. After spin coating, the samples were thermally annealed at 120 °C in air on a hotplate for 20 min. The P3HT layer thickness of the samples was measured using a KLA-Tencor α-Step IQ surface profiler.

### Photovoltage

Electrochemical measurements have been conducted using a potentiostat and an electrochemical cell in a three-electrode setup. The used potentiostat is a Metrohom PGSTAT302N. The reference electrode was a saturated Ag/AgCl (Metrohm 60733100) and the counter electrode was a Pt wire (Metrohm 60301100). Samples were connected using crocodile clips in direct contact with ITO over a portion of the sample not covered by P3HT. Films were covered with hole-punched black vinyl tape to expose only a circular 0.2 cm^2^ area of the sample to light. The used electrolytic solution was a 200 mM NaCl solution mixed with Milli-Q water and not pH-adjusted. The used cell was a Pine Research RRPG147 quartz photoelectrochemical cell. Oxygen concentration was measured using an electrochemical meter (VWR, DO 40-K) and reduced by continuously fluxing nitrogen in the cell. The illumination was supplied by a green LED (Thorlabs M530L3-C5, 530 nm, driven by a DC2200 LED driver). The LED was placed at 25 cm from the sample and operated at maximum power. The intensity of light hitting the sample was 500 W m^–2^. Light was allowed to fall from two directions: from the glass/ITO side, for simplicity accounted only as “ITO side”, or from the P3HT/electrolyte interface side, namely, the “electrolyte side”.

Photovoltage recordings were acquired operating the potentiostat in high-speed mode using a sample rate of 10^4^ s^–1^. Photovoltage curves are all zeroed to the open circuit potential value, which experimentally oscillates between 0.1 and 0.2 V vs saturated Ag/AgCl. The same has also been done for the simulations.

## Results and Discussion

### P3HT in Contact with the Electrolyte

**Figure 2:**
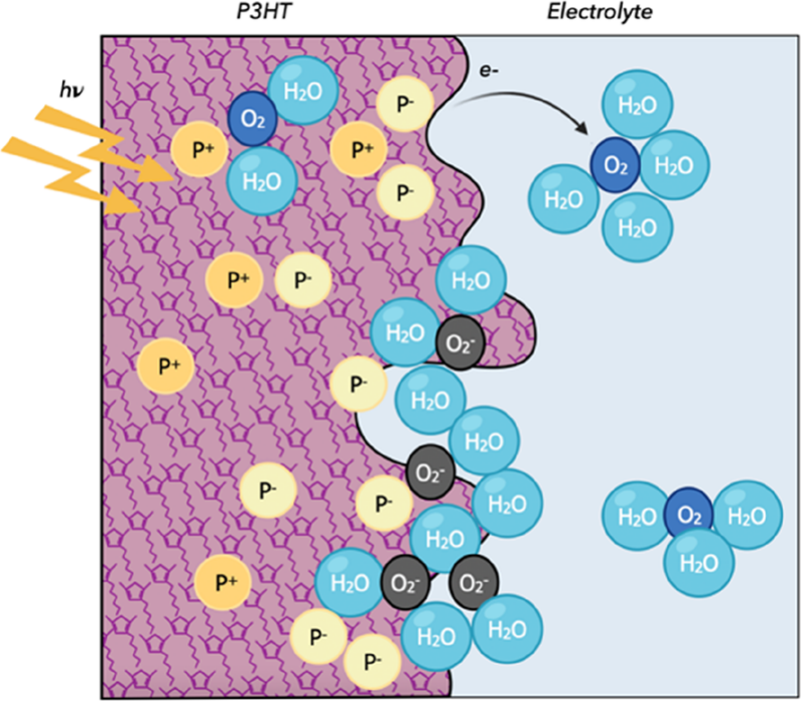
Faradic oxygen reaction at the P3HT/electrolyte interface and O_2_^–^ screening.

When P3HT films are kept in air or water, oxygen easily diffuses inside, leading to the formation of a complex with the polymer [P3HT^+^:O_2_^–^]. When in the presence of water, the complex is possibly hydrated, with a partial redistribution of the electronic charge and a larger electron affinity. The process occurs at a faster rate in the presence of light.^27,33,34^ It is reversible and it does not break the polymer conjugation, but it introduces electron trapping sites that essentially hamper electron mobility. Accordingly, after 24 h of immersion, the absorption spectrum of the polymer film remains essentially unchanged, while the photoluminescence is almost totally suppressed. This can be rationalized by assuming that photogenerated singlet states undergo energy transfer or charge transfer (with minor probability) to the oxygen complex that deactivates nonradiatively.^35^ While oxygen diffuses easily in the whole film volume, providing a source of p doping, the surface boundary presents some peculiarities. Here, free volume hosts water molecule clusters, there is a higher oxygen concentration, and important changes in morphology take place. The surface of the polymer in contact with the electrolyte gets less hydrophobic,^36^ possibly due to polymer swallowing. Water molecules polarize the polymer chains, causing a down shift in energy of the frontier orbitals in proximity of the aqueous solution.^28^ The size of the affected region, which corresponds to a diffuse interface, is estimated from geometrical consideration by measuring the capacitance of the film in contact with the electrolyte to be about 20 nm (Supporting Information Section S4).^35^ A graphical schematized description is reported in Figure 2. The energy shift resembles the band bending occurring in a p-type semiconductor. Even if the physics behind is rather different, the similarity holds true, as for instance regarding negative charge accumulation at the surface upon illumination. According to the p-type behavior, the illuminated polymer behaves as a photocathode that reduces the electrolyte species, predominantly oxygen but also hydrogen, possibly assisted by surface polymer protonation.^27,37,38^

### Photovoltage in Oxygenated Conditions

Here we describe the electrochemical response to illumination of P3HT layers grown on ITO-covered glass substrates. Measurements are done in a three-electrode configuration as reported in Figure 1. Experiments are carried out with the electrolyte solution in equilibrium with lab atmosphere.

All reported results were acquired ensuring the reproducibility of the PV curves.

**Figure 3:**
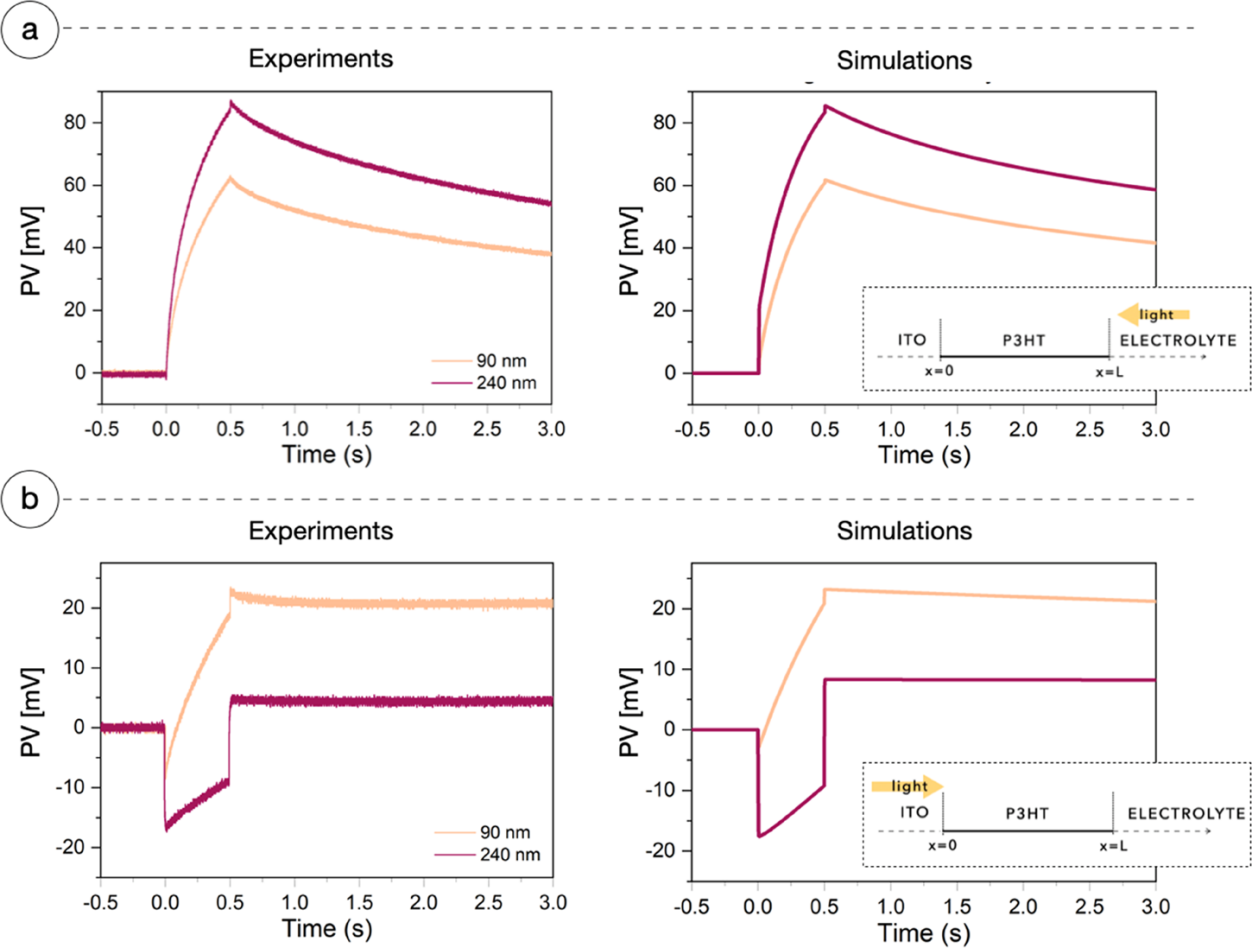
Comparison between experimental PV measurements (left) and simulations (right) on samples with different active layer thicknesses: 90 and 240 nm. Light is switched on between 0 and 0.5 s. Panel (a) reports the results obtained when the light stimulus impinges from the electrolyte side: the PV shows a positive shift. Panel (b) reports the results obtained when the light stimulus impinges from the ITO side: the PV shows an initial negative shift that slowly returns to positive values.

Figure 3 (left) shows the experimental PV curves for two thicknesses of the active layers. In panel (a), we illustrate the measurements corresponding to illuminating the sample from the P3HT side. Both samples show a positive PV peak that decays when the illumination is turned off. The dynamics are similar; however, the peak is 20 mV lower in the thin sample than in the thick one. In panel (b), the PV is recorded illuminating from the ITO side. For both samples, the PV abruptly drops to negative values and then starts showing a slow recovery. For the thicker sample, the PV initial drop is larger and its recovery is slower. Indeed, the PV signal never becomes positive during illumination, while in the thin layer PV reaches +20 mV. Once the illumination is stopped, at 500 ms, the PV becomes positive and very slowly decays to 0. The dependence of the photovoltage on the thickness of the active layer was also reported in Gautam et al.^39^ for bulk heterojunction systems and appears to be valid also for our configuration.

The right panels of Figure 3 show the simulated PV curves. The model simulates the photogeneration of “long-lived” charge carriers (polarons) in the bulk of the polymer and their evolution in time. We assume a small and homogenous photogeneration quantum efficiency (3.5 × 10^–4^), according to the well-established notion that in such polymer semiconductors most photoexcitations are neutral (namely, singlet excitons and polaron pairs) and short lived. Note that charge generation at the ITO interface is not necessary to reproduce the experiments. As discussed above, this can be explained by considering charge generation at π-stacking lamellae associated with the crystalline phase of RR-P3HT.^23^ In our case, the fraction of crystalline phase has been estimated to be about 40% using the model of Spano et al.^40−42^ (Supporting Information Section S3), also in agreement with the thickness-dependent estimation of Nádaždy et al.^43^ We model the unintentional doping due to the permanence of the sample in an oxygenated environment with an effective approach.^44^ The estimated doping concentration from our Mott–Schottky analysis is of the order of ∼10^23^ m^–3^ (Supporting Information Section S4). It is higher with respect to Choi et al.,^44^ probably due to the fact that before our analysis, we have performed 2 h of light soaking at high intensity. The doping process however does not seem to play a relevant role in enhancing the conductivity of the film, for we measured a conductivity of about 10^–5^ S cm^–1^, very small compared to the literature values (Supporting Information Section S5).^45^ Its major effect is rather electron trapping that essentially hampers n-transport. Accordingly, a realistic measure of electron mobility is not possible in our sample. All this leads us to consider a very larger asymmetry in mobility (p-type unipolar transport), and trap-assisted recombination. The recombination time has been estimated in agreement with the doping density measured from the Mott–Schottky, and are in agreement with MacKenzie et al.^46^ The use of SRH recombination has been demonstrated to well describe disordered organic semiconductor recombination phenomena, in which Langevin recombination appears to be negligible.^46,47^ As a result of model assumptions, charge carrier populations evolve according to their concentration and voltage gradients.

To describe the interface with the electrolyte, we introduce a process of pseudocapacitive charging of the wet surface driven by charge transfer toward dissolved oxygen,^27^ whose rate is described by the Marcus–Gerischer equation (eq 1). The presence of a diffuse capacitive-like interface is also supported by the comparison between the geometrical and interface capacitance. By geometrical considerations, we estimate the P3HT layer capacitance to be around 44 nF, while we estimate a 316 nF interface capacitance using Mott–Schottky data (Supporting Information Section S4). This allows us to estimate the width of the diffuse interface around 19 nm (*d* = *A*·ϵϵ_r_/*C*_in_). The diffuse interface region has a peculiar morphology because hydrated oxygen penetrating through the polymer chains induces swelling of the polymer film. This generates topological minima in the polymer free volume near the surface that can accommodate clusters of water molecules. Negative charge carriers trapped by reducing oxygen exert an electrostatic force on the water multipoles, whose polarization in turn generates a polaronic effect. The polaron binding energy is typically 0.5 eV. The latter has been indeed studied in other organic semiconductors both theoretically and experimentally.^48^ Water molecules screen the Coulombic field and facilitate the release of positive carriers following charge separation. Under concentration gradient, hole diffusive current spreads the positive charge away from the interface, contributing to the buildup of the electric field. In addition, both local charging and water penetration in the diffuse interface layer reduce the polymer surface hydrophobicity,^35^ modulating the adhesion properties. Finally, water polarization downshifts the energy of the superoxide, stabilizing it. Upon switching off light, the diffusion current reverses its sign and the positive polarons recombine with the negative ones.

The simulation reproduces well the experimental data and the qualitative trends in changing both the photoactive layer thickness and the illumination side. All adopted parameters and equations are reported in the experimental and supplement section (Supporting Information Sections S1 and S2). The emerging picture is the following: positive and negative charge carriers are photogenerated with an initial distribution in space which follows the Lambert–Beer profile. Holes quickly diffuse across the bulk, while electrons remain virtually fixed, due to trapping in chemical complexes that have a very low effective diffusion constant. Due to this asymmetry, the illuminated side initially shows an accumulation of negative charge, as discussed below, and the film gets electrically polarized.

### Role of the Illumination Side

**Figure 4:**
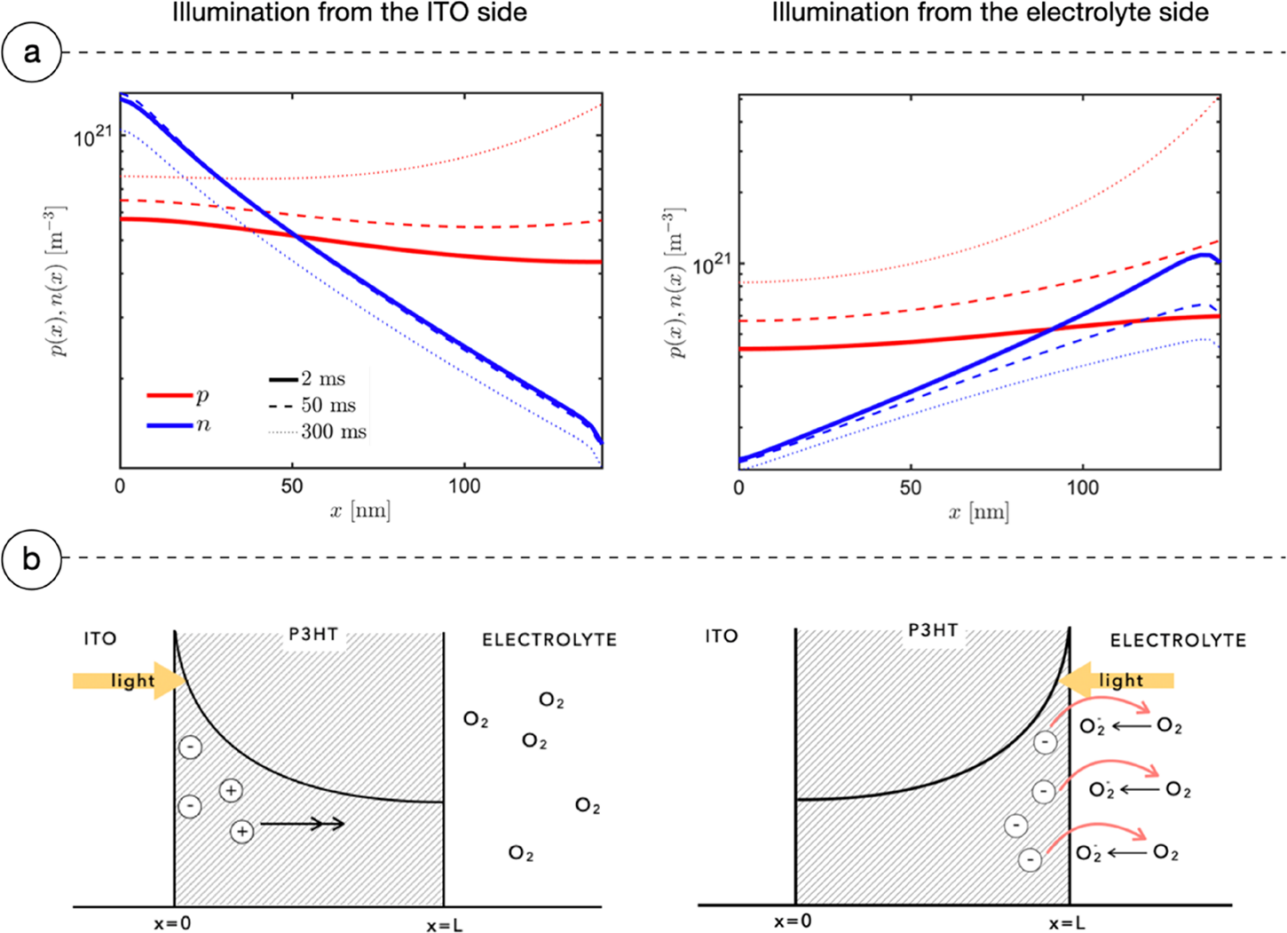
Simulation data and interpretation of charge carrier density redistribution. Panel (a), left, shows the number density of electrons and holes in the bulk of P3HT when light impinges from the ITO side. In panel (b), left, a scheme describes the physical interpretation. Due to the Lambert–Beer light profile, carriers have higher concentration near the ITO interface: due to the different mobility, holes quickly redistribute through the bulk of the material due to the diffusive flux, and electrons, instead, remain fixed where they have been generated, giving rise to an initial fast negative peak in the PV measures and simulations. Panel (a), right, shows the number density of electrons and holes in the bulk of P3HT when light impinges from the electrolyte side. Panel (b), right, shows its physical interpretation: in this configuration, electrons are highly available at the interface with the electrolyte to react with dissolved oxygen, thus positively charging the active material. In addition to this, holes instead can quickly diffuse toward the ITO interface. The two effects give rise to a positive PV.

**Figure 5:**
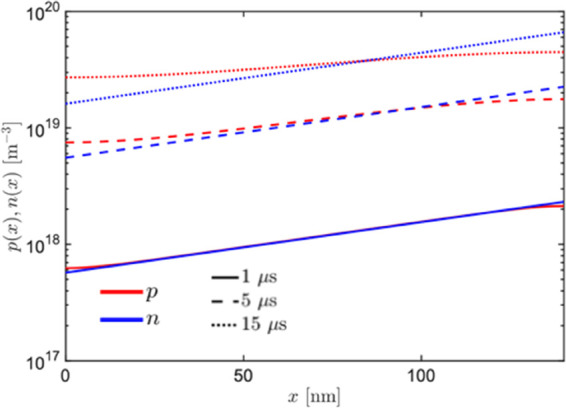
Hole and electron number densities across the bulk of the polymer when illuminated from the electrolyte side. The two curves show how the value of the number densities changes as a function of the illumination time: after 1 μs from switching on the light, the two distributions overlap; after 5 and then 15 μs, holes visibly redistribute across the bulk due to the diffusive effect.

Results shown in Figure 3 indicate that illumination from different sides of the sample changes the PV behavior. During the illumination from the ITO side, the PV initially bursts toward negative values, whereas, when illumination arrives from the P3HT side, there is a steady growth in PV. We can explain this different behavior by observing the distribution in space of the charge carriers generated in the two cases. Light intensity profile follows the Lambert–Beer exponential law inside P3HT (Figure 4, panel b). Thus, the side of illumination, mediated by the thickness and mobility, determines the “ruling” interface. Figure 4, panel a, shows the simulated distribution of charge carriers inside the P3HT layer after it is illuminated for 2, 50, and 300 ms from ITO and electrolyte sides. The electron density decreases away from the excitation side according to the initial Lambert–Beer profile (in logarithmic scale). Thanks to diffusive gradients instead, holes quickly spread across the bulk of the active layer, as visible in Figure 5. The reason for the different behavior is the difference in mobility between the two carriers. Note that we assumed that Einstein–Smoluchowski relation holds true, so mobility and diffusion coefficient are proportional. Holes are mobile, while electrons are trapped at the oxygen complexes.

This is accounted for by considering a strong difference in mobility and diffusion coefficient for the two carriers, as discussed above. Since an explicit value for the mobility of the electrons in this experimental condition is not available, it has been put equal to μ_n_ = 10^–12^ m^2^ V^–1^ s^–1^, namely, four orders of magnitude smaller than that of holes μ_p_ = 10^–8^ m^2^ V^–1^ s^–1^, in agreement with the physical picture reported in ref (49). Accordingly, electrons move less than 1 μm in 500 ms. The good agreement of the simulations with the experimental results validates our supposition. Due to the fast redistribution of holes across the bulk in 5–10 μs (see Figure 5), the excitation at the ITO side results almost instantaneously into an excess of negative charge at the ITO surface. However, at the electrolyte interface, electrons are slowly pumped out of the polymer (the exit of the electrons when the sample is illuminated from the ITO side induces a current *I* = 80–700 pA, depending on the thickness of the illuminated sample, which corresponds to about 10^8^–10^9^ electrons per second per device area). This reduces the negative PV, and in a thin sample can overturn the PV sign. When light impinges onto the electrolyte side, negative charge accumulates there efficiently and because electron transfer is superlinear with electron density (eq 1, see methods), it leads to the growing positive PV (in this case, the current induced by the exit of electrons is of the order of *I* = (3–4) nA or about 2 × 10^10^ electrons per second). The difference in thickness just affects the final PV value due to the different sample optical density.

### Role of Oxygen in P3HT–Electrolyte Interfacial Mechanisms

**Figure 6:**
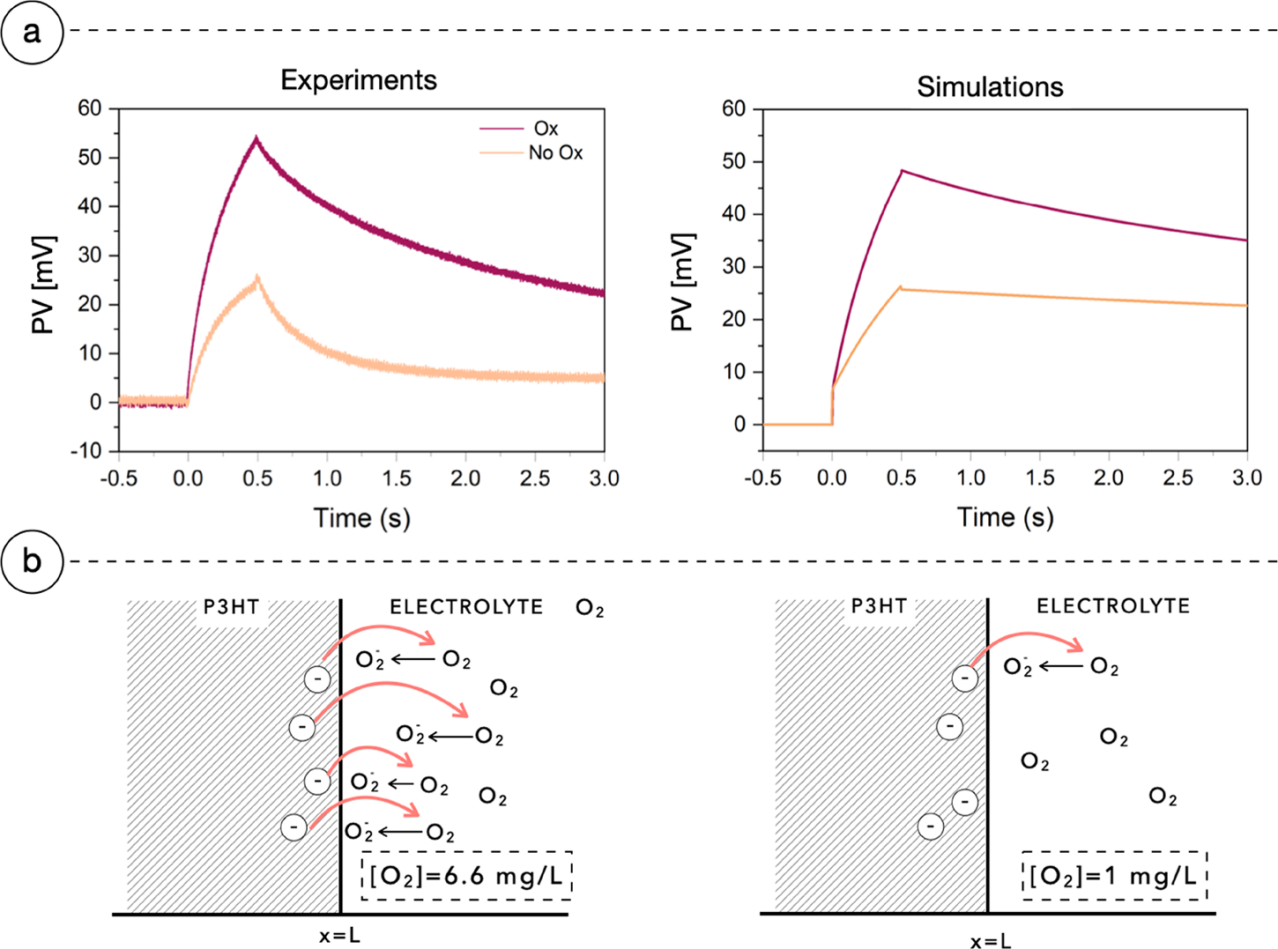
Faradic oxygen reaction at the P3HT/electrolyte interface and its effect on PV curves. Panel (a) shows the experimental PV measures (left) and PV simulation (right) varying the concentration of molecular oxygen dissolved in the electrolyte on a sample with 140 nm of the active layer. Light is switched on between 0 and 0.5 s and is impinging from the electrolyte side. The PV curves are decreasing in amplitude as the concentration of molecular oxygen is lowered. Panel (b) qualitatively shows how the different concentrations of molecular oxygen affect the exit of the electrons of the bulk and indirectly the amplitude of PV. In the two cases, the oxygen concentration is *c*_ox_ = 6.6 and 1 mg L^–1^, respectively.

The results of Figure 3 are obtained performing PV measurements with the electrolyte solution in equilibrium with lab atmosphere. In such conditions, the dissolved O_2_ concentration is 6.6 ± 0.1 mg L^–1^, measured with an O_2_ sensor. To prove that oxygen has a role, we repeated the electrochemical experiments by continuously fluxing nitrogen into the sealed electrochemical chamber, thus reducing the dissolved O_2_ concentration to 1.0 ± 0.1 mg L^–1^. Figure 6a left panel reports the PV of a sample under oxygenated and nonoxygenated conditions excited from the electrolyte side. At low oxygen concentration, the experimental results are qualitatively similar to those at the oxygenated ones, yet the PV peak value is halved. Upon illumination, the dissolved oxygen in solution accepts negative carriers from the P3HT, reducing to O_2_^–^.^27^ This phenomenon is associated with a reversible trapping^28^ occurring at the hydrated interface. P3HT is oxidized, and it loses electrons accumulating a positive net charge that makes the PV more positive. When light is switched off, the PV decays toward its initial equilibrium value due to bulk recombination of free charges and reversion of the trapping effect,^28^ described in the model as a surface recombination of holes with the O_2_^–^ accumulated during photostimulation. The surface recombination allows the P3HT to recover its initial voltage, retrieving the initial electroneutral conditions after a certain amount of time. The results of the simulation are reported in Figure 6a, right panel. They are conducted changing the value of *c*_ox_ in eq 1 and putting it equal to the two values measured experimentally for the oxygenated and nonoxygenated conditions. The kinetics of the two panels in Figure 6a shows indeed a subtle difference, being smoother in the experiment. This may be because in reality the parameter values are distributed, thus making the kinetics more dispersive. This does not occur in the simulation, where we assume single value parameters. Despite the small discrepancy, the time to peak of simulations coincides with the one found with the experiments, thus suggesting that the first-order phenomena are well captured by the equations of the model.

All of the curves have been obtained using the same value for the tunneling coefficient of electrons at the interface based on the Marcus–Gerischer model, namely, *k*_t_ = (7 ± 2) × 10^–30^ m^4^ s^–1^, in agreement with data reported in the literature.^30^ For hole surface recombination, we used *k*_p_ = (3.5 ± 2.5) × 10^–23^ m^3^ s^–1^. The results unequivocally indicate that oxygen is the major electron acceptor and P3HT is working as a photocathode, in accordance with the literature.^27,50−52^

Finally, a comment on the PV off-transient with light coming from the ITO side. We already stated that, as light is switched on, holes instantaneously redistribute across the bulk, thus leaving an accumulation of negative charge at the interface with the ITO. This gives rise to the abrupt negative peak observed in Figure 3b at *t* = 0. When light is switched off, recombination of the photogenerated carriers takes place on a time scale which is dictated by the time constant of the SRH recombination, namely, τ_n_ = τ_p_ = 0.1 ms,^46^ leaving an excess of positive charge in the polymer due to the escape of electrons toward the electrolyte. We also notice that the PV recovery toward equilibrium is slower (Figure 3b) with respect to when light arrives from the electrolyte side (Figure 3a).

According to our model, PV recovery is mainly driven by the recombination of holes at the electrolyte interface, described by the following equation
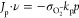
2*p* is the number density of holes at *x* = *L*, σ_O_2_^–^_ is the negative surface charge density given by the accumulated O_2_^–^ charges at the interface, and *k*_p_ is the time constant of the reaction (Supporting Information Section S2). O_2_^–^ is indeed supposed to accumulate at the interface, with only a minor negligible fraction evolving into peroxide: this is allowed by the polaronic effect of hydrated oxygen, which screen the O_2_^–^ and allow them to remain stable at the interface. In diluted charge conditions indeed, the second reduction of O_2_^–^ is assumed to be not statistically favored. Intercalation of anions in the bulk, retained responsible for electroneutrality recovery in certain experimental conditions,^44^ has been excluded from PV measurements with a high steric hindrance electrolyte, as shown in Supporting Information Section S7. Therefore, we assume that the main phenomenon responsible for electroneutrality restoration is the reversible detrapping of electrons from oxygen complexes. The physical picture of the diffused interface with the electrolyte is schematized in Figure 2.

When light comes from the ITO side, fewer electrons exit the bulk of P3HT to reduce the molecular oxygen, thus leading to a lower concentration of negative charges at the interface (small σ_O_2_^–^_); therefore, a lower *J*_p_ and a slower recovery of the initial voltage conditions can be observed.

### Close Box Simulations

To better understand the role of the different parameters in the model, we present here ad hoc simulations that run under nonphysical conditions.

To single out what happens inside the bulk of the semiconductor when no reaction occurs at the interface, we knocked-out electron transfer toward the electrolyte. Computationally, this translates into forcing zero current at the semiconductor–electrolyte interface, hence respecting the following conditions
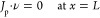
3
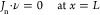
4where *J*_p_, *J*_n_, and *ν* are the hole current density, electron current density and outward unit vector normal to the surface, respectively.

**Figure 7:**
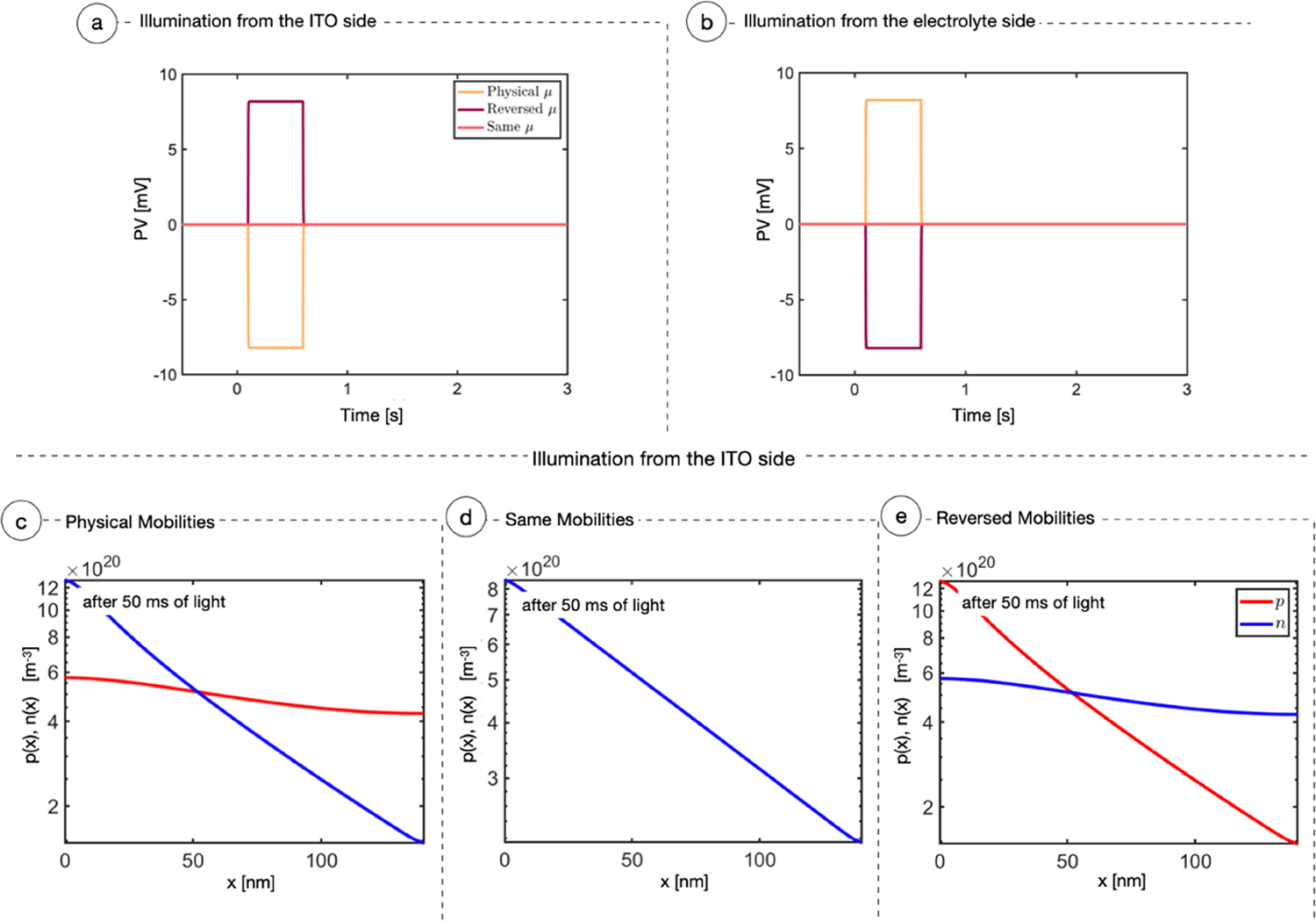
Mobility study. Panels (a) and (b) show the simulations of PV curves assuming no interface mechanisms occurring at the electrolyte side (*J*_n_ and *J*_p_ both are equal to zero) in three different configurations: (i) with physical mobilities, namely, μ_p_ = 10^–8^ and μ_n_ = 10^–12^ m^2^ V^–1^ s^–1^; (ii) with equal mobilities μ_p_ = μ_n_ = 10^–12^ m^2^ V^–1^ s^–1^, where no PV effect is appreciable due to electroneutrality; (iii) with reversed mobilities, namely, μ_p_ = 10^–12^ and μ_n_ = 10^–8^ m^2^ V^–1^ s^–1^, where the PV curves appear flipped with respect to the physical mobility case. Panels (c), (d), and (e) show the charge carrier number densities after 50 ms of light across the bulk with light coming from the ITO side in scenarios (i), (ii), and (iii), respectively.

Figure 7a,b reports the PV of a 140 nm thick P3HT layer simulated with no currents exiting toward the electrolyte. The orange curve shows the case in which mobilities have been kept as the physical value, used for previous simulations. Any other parameter is unchanged. As the sample is illuminated from the ITO side (Figure 7a), the PV suddenly drops to −7 mV and stays constant until light is turned off. The charge distributions in Figure 7c clearly correlate with the observed PV. As discussed above, positive charges tend to immediately distribute across the P3HT layer, while electrons essentially maintain the initial Lambert–Beer profile. This causes the film polarization with excess negative charge near the illuminated side and positive charge on the far side. When light hits the electrolyte side, the scenario in Figure 7c is reversed with respect to the *x*-axis: at the ITO surface, there is an excess of positive charge that induces positive photovoltage (Figure 7b, orange curve).

To test the role of the asymmetry in carrier mobilities, we ran the model using the same mobility for both carriers. We obtained the results of Figure 7a,b (pink curves) and Figure 7d. The charges generated inside the P3HT maintain the Lambert–Beer profile (Figure 7d), while the system electroneutrality (both globally and locally) generates no PV.

Finally, we tested what would happen reversing the values of the mobilities: μ_n_ = 10^–8^ m^2^ V^–1^ s^–1^ and μ_p_ = 10^–12^ m^2^ V^–1^ s^–1^. In these conditions, we observe that both the charge distributions (Figure 7e) and the PV are the opposite of the real case (Figure 7a,b, purple curves).

**Figure 8:**
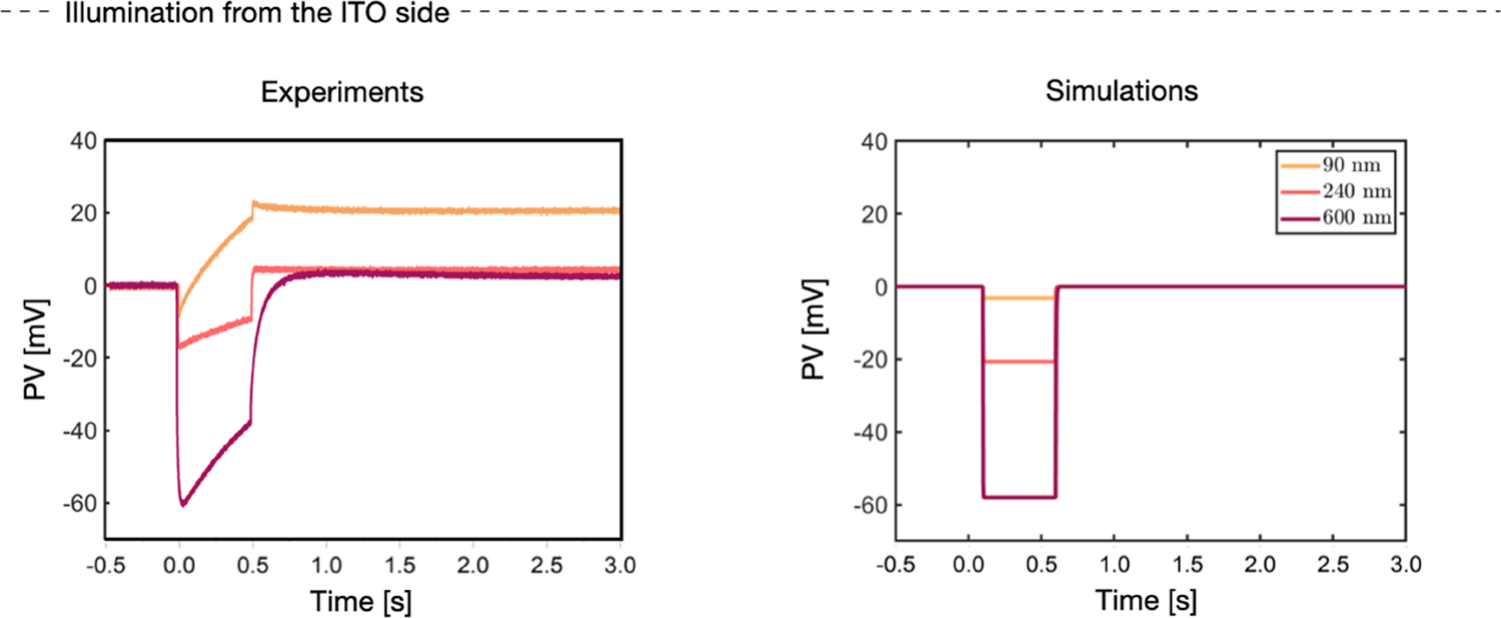
Two panels compare the experimental PV curves (on the left) obtained illuminating samples of different thicknesses from the ITO side with the simulations of PV curves (right) with all of the interface mechanisms switched off. The curves are shown for a sample with different values of the active layer thickness, namely, 90, 240, and 600 nm, respectively.

Comparing PV experiments performed on samples with increasing thickness and exciting the ITO side allows modulating the contribution of the reactions at the electrolyte side. Experimental data in Figure 8 left panel show that the initial negative PV gets larger as the thickness increases, while the change in sign of the PV only occurs for the thinnest film. This is consistent with the model we propose above that explains the recovery toward positive PV as due to the electron transfer at the electrolyte interface. The simulations, Figure 8 right panel, ran without including such transfer account well for the nonlinear dependence of the initial negative plateau with the thickness and directly relate them to diffusive effects (Supporting Information Section S8).

## Conclusions

In this article, with the combined use of mathematical modeling and electrochemistry, we examined the behavior of a P3HT film sandwiched between an ITO substrate and a saline electrolyte under light excitation. The system is considered as the simplest approximation to the abiotic/biotic interface where the polymer film is interfaced with living cells. With this approach, we were able to identify the fundamental phenomena occurring at the hybrid interface and to quantitatively characterize them.

The dynamics at the interface of P3HT with a 0.2 M NaCl electrolyte is characterized by two main mechanisms: a pseudocapacitive coupling due to electron transfer toward oxygen dissolved in the electrolyte solution and a surface recombination of holes (positive polarons) with the negative charge accumulated there. In addition to this, and therefore independently from the solution in which the P3HT is immersed, under illumination the polymer film is electrically polarized by the asymmetric charge carrier diffusion. The latter is described considering both the concentration gradient and the Coulombic interaction by the diffusion–drift transport equations coupled with the Poisson equation.

The quantitative description of the electrostatics of the interface gives us some hint in the interpretation of the mechanisms occurring at the in vivo interface of the recently tested retinal prosthesis in animal models,^10,35^ a natural highly complex system. The polymer film is in contact with retinal cells through a complex layer of proteins and any other biological components that are protruding out of the cell membrane to fill the cleft. Note that this does not hamper oxygen to be available at the polymer surface, while it certainly reduces the electrical conductivity with respect to the free electrolyte. As our results suggest, the diffusion–drift dynamics and the negative surface charging driven by oxygen may be suspected to be responsible for the interaction, possibly leading to modifications of the cleft environment that in turn might be sensed by the cell membrane. Furthermore, we expect that the reduced circulation in the cleft could assist the pseudocapacitive charging of the interface. The incoming light defines the sign of electrical polarization, with negative charging of the illuminated surface with respect to the opposite one. This gives us a further hint about the possible phenomena responsible for the biohybrid interaction: the negative charge region can spread through the cleft, mainly filled by proteins, causing depolarization of the membrane voltage. In addition, the highly irregular polymer surface topology may be the site of “hot” spots, where strong electric fields are generated by charge accumulation, possibly increasing the depolarization currents through the cell membrane.

## Supporting Information Available

Drift–diffusion model description, estimate of the crystalline component, Mott–Schottky curves, conductivity measures, photovoltage with different electrolytes, and additional simulation results (PDF)
