# Correction to “Photoelectrochemistry and Drift–Diffusion Simulations in a Polythiophene Film Interfaced with an Electrolyte”

**DOI:** 10.1021/acsami.2c06695

**Published:** 2022-05-02

**Authors:** Greta Chiaravalli, Giovanni Manfredi, Riccardo Sacco, Guglielmo Lanzani

The authors would like to add the section “Acknowledgements”.

