# Supplementary material for: Photoelectrochemistry and Drift–Diffusion Simulations in a Polythiophene Film Interfaced with an Electrolyte
Source: ACS Appl Mater Interfaces. 2021 Jul 26;13(30):36595–604. doi: 10.1021/acsami.1c10158 (PMC8397247; doi:10.1021/acsami.1c10158)
Supplement: Supplementary file 1 — am1c10158_si_001.pdf [file am1c10158_si_001.pdf]

Supporting Information: Photoelectrochemistry and Drift-Diffusion Simulations in a Polythiophene Film Interfaced with an Electrolyte

Greta Chiaravalli[1,2], Giovanni Manfredi[1], Riccardo Sacco[3], and Guglielmo Lanzani*[1,2]

[1]Center for Nano Science and Technology, Istituto Italiano di Tecnologia, 20133 Milan, Italy

[2]Department of Physics, Politecnico di Milano, Milan, Italy.

[3]Department of Mathematics, Politecnico di Milano, Milan, Italy.

Corresponding author address: Guglielmo.Lanzani@iit.it

1 Drift Diffusion model

To mathematically describe the physical mechanisms that characterize the microscopic properties of the P3HT bulk, we propose an adapted version of the time-dependent Drift Diffusion model (DD), in which the classic formulation used in the study of contemporary semiconductor devices in micro and nanoelectronics is suitable modified to effectively simulate the nonstandard experimental conditions conducted in our work [1–4]. The set of equations comprises two continuity equations for the free carriers, holes and electrons, and the Poisson equation for the electric field. The photoconversion of incident light into excitons and their successive transport throughout the P3HT bulk is, instead, taken into account with a lumped approach by performing suitable modeling approximations.

1.1 Geometry

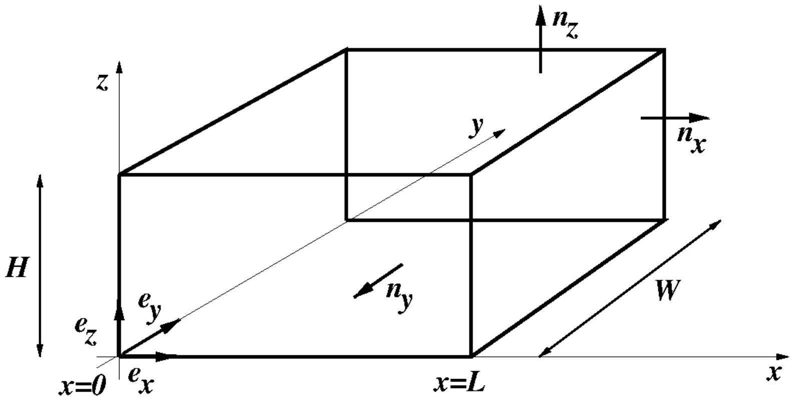

Figure S 1: Schematic representation of the simulated device. The unit vectors of the three coordinate axes are denoted by $\mathbf{e}_x$, $\mathbf{e}_y$ and $\mathbf{e}_z$ whereas the unit normal vectors on the boundary of the device are $\mathbf{n}_x = \mathbf{e}_x$, $\mathbf{n}_y = -\mathbf{e}_y$ and $\mathbf{n}_z = \mathbf{e}_z$

The device object of our analysis and simulation is schematically represented in Figure S 1 . The P3HT bulk is a three-dimensional (3D) parallelepiped with length L, width W and height H. The interface between the P3HT bulk and the ITO layer is located at $x = 0$ whereas the interface between the P3HT bulk and the electrolyte solution is located at $x = L$. In the remainder of this text we assume that photogeneration mechanisms, carrier transport and electric effects happen solely along the x direction and do not depend on the other spatial coordinates y and z. We also denote by Ω the one-dimensional (1D) open interval $(0, L)$ that represents the computational domain used for device modeling and simulation whereas $T > 0$ is the length of the time integration interval.

1.2 Time Domain

In order to describe the time evolution of the electrochemical experiment, we are solving our system of equations into a time interval of 3.1 seconds, with $t \in (0, T = 3.1\,\mathrm{s})$. Light is switched on at $T_i = 0.1$ s and off after $T_f = 0.5$ s, simulating a light stimulus of the same duration of the one of the experiment. The PV curves reported in the article have been rigidly shifted to match the switch on light time of the experiment.

1.3 Excitons

The excitation phenomena occurring as a consequence of light absorption by the P3HT layer of our device are described by a parabolic problem. We refer here to excitons to mean the initial neutral, diffusing, singlet photoexcited states, disregarding their exact nature. Their diffusion constant is an effective value due to the combination of coherent motion in the crystalline regions and incoherent motion in the amorphous region.

$$\frac{\partial X}{\partial t} + \nabla_x \cdot \mathbf{J}_X = G_{\text{light}}(x,t) - k_{\text{rec}}X - k_{\text{diss}}X \qquad \text{in } \Omega \times (0,T) \qquad (1a)$$

$$\mathbf{J}_X = -D_X \frac{\partial X}{\partial x}\mathbf{e}_x \qquad \text{in } \Omega \times (0,T) \qquad (1b)$$

$$X(x,0) = 0 \qquad \text{in } \Omega \text{ at } t=0 \qquad (1c)$$

$$\mathbf{J}_X \cdot \mathbf{n}_x = 0 \qquad \text{at } x=0,\ L \times (0,T) \qquad (1d)$$

where $\nabla_x(\cdot) = \partial(\cdot)/\partial x$. Equation (1a) expresses the fact that the time rate of change of exciton density equals the net balance between the diffusive flux ($\nabla_x \cdot \mathbf{J}_X$) and the net production rate due to the difference between a source term (G_{light}) and a sink term ($k_{\text{rec}}X + k_{\text{diss}}X$). As light impinges onto the organic substrate, it creates neutral excited states, namely excitons. Exciton flux $\mathbf{J}_X$ is defined according to Fick's law (1b), D_X being exciton diffusivity, since these states do not have a net charge and hence the electric field does not determine a drift on them. The source term G_{light} represents the exciton generation rate due to light absorption. The quantities $k_{\text{rec}}X$ and $k_{\text{diss}}X$ represent instead the rate constants for the transition of excitons back to the ground state or their dissociation into a couple of free charge carriers. The remaining two relations (1c) and (1d) are the initial condition and the boundary conditions, respectively. In particular, Eq. (1c) expresses the fact that no excitons are present inside the P3HT bulk before light illumination, whereas Eq. (1d) expresses the fact that all the excitons that are photoexcited inside the P3HT bulk *remain* within the bulk and are not allowed to flow out the device across the boundary interfaces.

- **Assumption 1**: We set

$$\frac{\partial X}{\partial t} = 0 \qquad x \in \Omega,\ t \in (0,T) \qquad (2a)$$

Assumption 1. is consistent with the fact that the photoconversion dynamics

is almost instantaneous so that the time derivative of exciton density may be neglected over the time scale of the phenomena of interest in our application.

- **Assumption 2**: We set

$$\nabla_x \cdot \mathbf{J}_X = 0 \qquad x \in \Omega,\ t \in (0,T) \tag{2b}$$

Assumption 2. implies that $\mathbf{J}_X$ is spatially constant which, due to the boundary conditions (1d), yields $\mathbf{J}_X(x,t) = 0$ for all $x \in \overline{\Omega}$ and $t \in (0,T)$.

Using (2) into (1) we obtain

$$0 = G_{\text{light}}(x,t) - k_{\text{rec}}X(x,t) - k_{\text{diss}}X(x,t)$$

from which we get

$$X(x,t) = \frac{G_{\text{light}}(x,t)}{k_{\text{rec}} + k_{\text{diss}}} \qquad x \in \Omega,\ t \in (0,T) \tag{3}$$

1.4 Free carriers

The continuity equations for holes and electrons can be written as:

$$q\frac{\partial p}{\partial t} + \nabla_x \cdot \mathbf{J}_p = q\left(k_{\text{diss}}X - \frac{pn - n_i^2}{\tau_n(p+n_i) + \tau_p(n+n_i)}\right) \qquad \text{in } \Omega \times (0,T) \tag{4a}$$

$$\mathbf{J}_p = -q\mu_p p\frac{\partial \psi}{\partial x}\mathbf{e}_x - qD_p\frac{\partial p}{\partial x}\mathbf{e}_x \qquad \text{in } \Omega \times (0,T) \tag{4b}$$

$$q\frac{\partial n}{\partial t} - \nabla_x \cdot \mathbf{J}_n = q\left(k_{\text{diss}}X - \frac{pn - n_i^2}{\tau_n(p+n_i) + \tau_p(n+n_i)}\right) \qquad \text{in } \Omega \times (0,T) \tag{5a}$$

$$\mathbf{J}_n = -q\mu_n n\frac{\partial \psi}{\partial x}\mathbf{e}_x + qD_n\frac{\partial n}{\partial x}\mathbf{e}_x \qquad \text{in } \Omega \times (0,T) \tag{5b}$$

In the above equation systems, q is the elementary charge (C) and μ_p and μ_n are the carrier electric mobilities ($\text{m}^2\text{V}^{-1}\text{s}^{-1}$). The diffusion coefficients D_p and D_n (m^2s^{-1}) can be obtained by applying the Einstein-Smoluchowski relation

$$D_\nu = \mu_\nu V_{th} \qquad \nu = p,\, n$$

V_{th} being the thermal voltage.

Similarly to the exciton balance equation (1a), equation (4a) expresses the fact that the time rate of change of hole density equals the net balance between the drift-diffusive flux ($\nabla_x \cdot \mathbf{J}_p$) and the net production rate due to the difference between a generation term

$$G = k_{\text{diss}} X + \frac{n_i^2}{\tau_n(p + n_i) + \tau_p(n + n_i)}$$

and a recombination term

$$R = \frac{pn}{\tau_n(p + n_i) + \tau_p(n + n_i)}$$

The same considerations apply to equation (5a).

Interestingly, the effect of model reduction of the exciton system (1) performed in Section 1.3 reflects into a coupling between the excitonic mechanism and the free carrier densities that is mathematically represented through the following *effective light generation term*

$$G_{\text{light, eff}} = \frac{k_{\text{diss}}}{k_{\text{rec}} + k_{\text{diss}}} G_{\text{light}}(x, t) \tag{6}$$

- **Assumption 3**: we assume k_{rec} and k_{diss} to be constant. In particular, the dependence of k_{diss} from the electric field, tipically described with the Braun-Onsager theory, is assumed to be negligible. This is justified by the evidence that, with an electric field of the order of 10^5 Vm^{-1}, typical of our device, the percentage correction due to the Braun-Onsager theory turns out to be of the order of 1%.

Therefore, we can retrieve

$$\eta_{\text{diss}} = \frac{k_{\text{diss}}}{k_{\text{rec}} + k_{\text{diss}}} \tag{7}$$

which exactly corresponds to the net charge generation efficiency introduced in Braun's theory [5, 6]. In our simulations, η_{diss} is set equal to $3.5 \cdot 10^{-4}$, in agreement with characteristic values for organic semiconductors. Assuming $k_{\text{rec}} \simeq \dfrac{1}{500 \text{ ps}} \simeq 2 \cdot 10^9 \text{ s}^{-1}$, we obtain that k_{diss} is equal to $7 \cdot 10^5 \text{ s}^{-1}$, a value in good agreement with existing data [6].

The term $G_{\text{light}}(x, t)$ mathematically describes how light impinges on the P3HT bulk. The dependence of $G_{\text{light}}(x, t)$ on the time variable is described through a step

function which is different from zero only in the 500 ms between T_i and T_f, defined in section 1.2.Therefore, when light is switched off, $G_{\text{light}}(x,t) = 0$, otherwise, when light is turned on, $G_{\text{light}}(x,t)$ depends on the incoming light direction. The dependence on the spatial variable is described through the Lambert-Beer relation, according to the following law:

$$\begin{cases} G_{\text{light}}(x,t) = I_0 \alpha \exp(-\alpha x) & \text{from ITO side} \\ \\ G_{\text{light}}(x,t) = I_0 \alpha \exp(-\alpha(1-x)) & \text{from electrolyte side} \end{cases} \tag{8}$$

where I_0 is the light intensity measured in (photons $\text{m}^{-2}\text{s}^{-1}$) and α is P3HT absorption coefficient (m^{-1}). All the values are reported in Table S 1.

1.5 The recombination-generation mechanism

In our model, the dynamics of free carriers within the P3HT layer are mathematically described by the two parabolic problems (4)- (5). According to these problems, free carrier balance takes into account generation mediated by the excitons and a net recombination described with a Shockley-Read-Hall (SRH) net recombination rate. Langevin recombination is instead assumed to be negligible. The SRH model describes a recombination-center mediated recombination of the two carriers, which takes effectively into account the trapping mechanisms related to the oxygen-P3HT complex. The net rate of recombination, denoted by $U = R - G$, can be written as a balance between spontaneous generation and recombination processes, deriving from a deviation from thermodynamical equilibrium condition, i.e.,

$$U = \frac{pn - n_i^2}{\tau_n(p + n_i) + \tau_p(n + n_i)} \tag{9}$$

where τ_n and τ_p are the carrier lifetimes and are assumed to be both equal to 10^{-4} s^{-1}, in agreement with the values for recombination reported in MacKenzie et al. [7].

1.6 Electric field

The dependence of the electric potential ψ and electric field $\mathbf{E}$ on the space charge density in the device is described by the Poisson equation:

$$\nabla_x \cdot \mathbf{D} = q\,(p - n + N_D - N_A) \qquad \text{in } \Omega \times (0,T) \tag{10a}$$

$$\mathbf{D} = \varepsilon \mathbf{E} \qquad \text{in } \Omega \times (0,T) \tag{10b}$$

$$\mathbf{E} = -\frac{\partial \psi}{\partial x}\mathbf{e}_x \qquad \text{in } \Omega \times (0,T) \tag{10c}$$

where $\mathbf{D}$ is the electric displacement vector, ϵ is the dielectric constant of P3HT (Fm^{-1}), $N_D - N_A$ is the net doping of the material (m^{-3}), N_D being the number density of donor atoms and N_A the number density of acceptor atoms. In our model the net doping has been neglected, consistently with the experimental measures on electric conductivity in dark conditions.

2 Boundary conditions

ITO interface ($x = 0$) The boundary conditions for the Poisson and continuity equations must satisfy the constraint on the total electric current experimentally set equal to zero. This is mathematically represented by the following relation

$$\mathbf{J}_{tot} = \mathbf{J}_n + \mathbf{J}_p + \frac{\partial \mathbf{D}}{\partial t} = 0 \tag{11}$$

From (11) we derive the value of the electric displacement as:

$$\frac{\partial \mathbf{D}(x,t)}{\partial t} = \mathbf{J}_{tot}(x,t) - \mathbf{J}_n(x,t) - \mathbf{J}_p(x,t) \tag{12a}$$

$$\int_0^t \frac{\partial \mathbf{D}(x,t)}{\partial t} dt = \int_0^t \left(\mathbf{J}_{tot}(x,t) - \mathbf{J}_n(x,t) - \mathbf{J}_p(x,t)\right) dt \tag{12b}$$

$$\mathbf{D}(x,t) = \mathbf{D}(x,0) + \int_0^t \left(\mathbf{J}_{tot}(x,t) - \mathbf{J}_n(x,t) - \mathbf{J}_p(x,t)\right) dt \tag{12c}$$

This relation holds for all $x \in \Omega$ and can be used to obtain a pointwise relation for the electric displacement at the ITO interface ($x = 0$). The value of $\mathbf{J}_{tot}$ is an input datum, experimentally set equal to zero, while $\mathbf{J}_n$ and $\mathbf{J}_p$ are iteratively computed by the numerical algorithm. In the experimental conditions of our interest, $\mathbf{J}_n$ and $\mathbf{J}_p$ are

supposed to be very small and have been set equal to 0. At the same time, $\mathbf{D}(x, 0)$ for all $x \in \overline{\Omega}$, when no light is impinging onto the P3HT layer, is supposed to be equal to zero, consistently with the fact that it represents the electric field between the ITO and the P3HT, a material which, when not illuminated, has similar properties to those of insulating materials. Therefore, we can write:

$$\mathbf{D}(0,t) \cdot \mathbf{n} = 0 \qquad t \in (0,T) \tag{13}$$

$$\mathbf{J}_p(0,t) \cdot \mathbf{n} = 0 \qquad t \in (0,T) \tag{14}$$

$$-\mathbf{J}_n(0,t) \cdot \mathbf{n} = 0 \qquad t \in (0,T) \tag{15}$$

Electrolyte interface ($x = L$) At $x = L$ we set

$$\psi(L,t) = V_{OCP} \qquad t \in (0,T) \tag{16}$$

where V_{OCP} is the constant value of the electric potential equal to the experimentally measured open circuit potential (OCP) vs Ag/AgCl KCl sat. The boundary condition (16) is consistent with the fact that, since illumination time is very short (500 ms), the system does not move too far from the equilibrium value of the OCP measured in dark conditions. Concerning the boundary conditions for the free electric charges, we suppose to have a net current of electrons exiting the bulk of P3HT due to a reaction of reduction of oxygen

$$O_2 + e^- \rightarrow O_2^-$$

This effect may be mathematically described by the Marcus-Gerischer theory. Holes instead are supposed to recombine with the reduced oxygen accumulating at the interface after illumination. This second process is described through a surface recombination model.

- **Electrons** To describe what happens to the negative free charges at the interface with the electrolyte, we adopt the Marcus-Gerischer theory; this theory is typically applied to quantitatively describe the heterogeneous kinetics of a reaction at the interface between an electrode and electrolyte, focusing on the overlapping of the electronic states of the two. A formal description of the total current density is approximately equal to the cathodic current density J_{cat} based on the Marcus-Gerischer model [8]. According to this theory, electron transfer is

proportional to the overlap of the empty oxidized states in the solution with the occupied energy levels for the electrons in the electrode. As a consequence, an electron is energetically favored to move from the P3HT into the solution, in order to reduce the species contained there and to generate a new oxidized state.

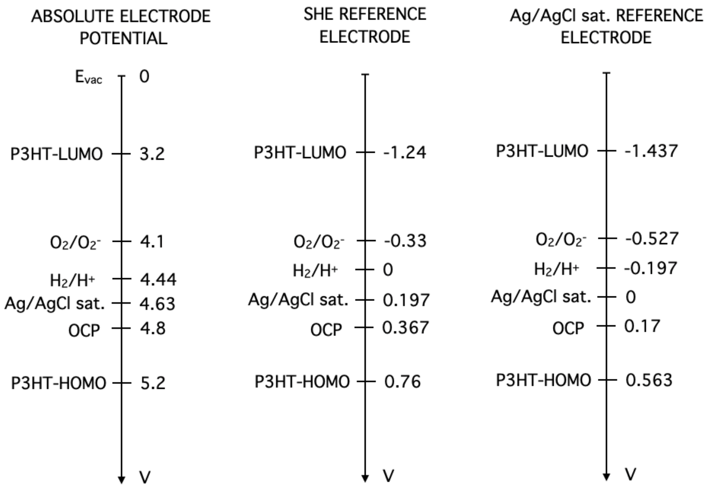

Figure S 2: The figure describes various reference configurations used to describe the potential levels of the system. The absolute electrode potential E^{abs} is bond to the electrochemical potential vs SHE through the relation $E^{abs} = E^{\mathrm{SHE}} + 4.44\ (\pm 0.02)$ V

Inorganic semiconductors In the case of inorganic materials, the cathodic current can be written upon assuming that the density of occupied states in the conduction band is equal to the density of free electrons in the conduction band, $n = f(\mathcal{E})g_{el}(\mathcal{E})$, where n is the electron density at the surface [8]

$$J_{cat} = \frac{q k_t c^{ox} N_A}{(\pi k_B T \lambda)^{1/2}} \int_{\mathcal{E}_c}^{+\infty} n \exp\left(-\frac{(\mathcal{E} - \mathcal{E}_F^{OX} - \lambda)^2}{4\lambda k_B T}\right) d\mathcal{E} \tag{17}$$

where n is the electron number density at $x = L$ and c_{ox} ($\mathrm{mol\,m^{-3}}$) is the concentration of molecular oxygen dissolved in the electrolyte. $\mathcal{E}_F^{OX}$ is the energy corresponding to the potential of the reaction of reduction of oxygen, as computed in Eq. (26), k_t is the transmission coefficient ($\mathrm{m^4\,s^{-1}}$) varying in the interval $[0, 1]$, 1 being is the typical value for adiabatic reactions and λ is the reorganization energy of the redox electrolyte (eV). The integral in (17) is typically evaluated

by making some approximations, as follows. Since the overlap between energy states on both sides of the interface is limited to a rather small energy range, the electron transfer may be assumed to occur mainly within 1 k_BT at the edge of the conduction band. Using this approximation, the integral can be replaced by inserting $d\mathcal{E} = 1k_BT$ and $\mathcal{E} = \mathcal{E}_c$ (see [8]). By doing so we obtain

$$J_{cat} = qk_tc^{ox}N_A\frac{k_BT}{(\pi k_BT\lambda)^{1/2}}\,n\exp\left(-\frac{(\mathcal{E}_c - \mathcal{E}_F^{OX} - \lambda)^2}{4\lambda k_BT}\right) \tag{18}$$

Organic semiconductors The description illustrated in the case of inorganic materials can be applied to organic semiconductors as follows. We assume the conduction band to correspond to the LUMO energy level and that the energy range in which electron transfer occurs is equal to a quantity σ to be specified later. In this way we obtain a cathodic current of the form

$$J_{cat} = qk_tc^{ox}N_A\frac{\sigma}{(\pi k_BT\lambda)^{1/2}}\,n\exp\left(-\frac{(\mathcal{E}_L - \mathcal{E}_F^{OX} - \lambda)^2}{4\lambda k_BT}\right) \tag{19}$$

The above expression can be used to define the following Robin-type boundary condition at $x = L$ for the electron density

$$-\mathbf{J}_n(L,t)\cdot\mathbf{n} = \alpha_n(L,t)n(L,t) - \beta_n(L,t) \tag{20}$$

where:

$$\alpha_n(L,t) = \frac{qk_tc^{ox}N_A}{(\pi k_BT\lambda)^{1/2}}\exp\left(-\frac{(\mathcal{E}_c - \mathcal{E}_F^{OX}(t) - \lambda)^2}{4\lambda k_BT}\right) \qquad t\in(0,T) \tag{21a}$$

$$\beta_n(L,t) = 0 \qquad t\in(0,T) \tag{21b}$$

- **Holes** Due to the experimental conditions of zero total current, the reduced oxygen produced at the interface is supposed to accumulate at the surface and to provide a recombination center for the holes flowing from the P3HT towards the interface. This also allows us to recover the electroneutrality of the whole polymer. The quantity $J_p(L,t)$ that exits the bulk is described through a surface recombination term

$$J_p(L,t) = -k_p\sigma_s(t)p(L,t) \tag{22}$$

where k_p is a time constant ($\mathrm{m^3\,s^{-1}}$), σ_s is the negative surface charge accumulated at $x = L$ ($\mathrm{C\,m^{-2}}$) and $p(L,t)$ is the hole number density at the electrolyte ($\mathrm{m^{-3}}$). The above expression can be used to define the following Robin-type boundary condition at $x = L$ for the hole density

$$\mathbf{J}_p(L,t) \cdot \mathbf{n} = \alpha_p(L,t)p(L,t) - \beta_p(L,t) \tag{23}$$

where:

$$\alpha_p(L,t) = -k_p\sigma_s(t) \qquad t \in (0,T) \tag{24a}$$

$$\beta_p(L,t) = 0 \qquad t \in (0,T) \tag{24b}$$

In order to compute the quantity $\alpha_p(L,t)$ we need to solve the following Cauchy problem for the total surface charge accumulating at the interface:

$$\frac{d\sigma_s(t)}{dt} = \mathbf{J}_n(L,t) \cdot \mathbf{n}|_{x=L} + \mathbf{J}_p(L,t) \cdot \mathbf{n}|_{x=L} \tag{25a}$$

$$\sigma_s(0) = 0 \tag{25b}$$

From the evaluation of σ, one can retrieve the concentration of reduced oxygen by assuming a diffusion thickness of the layer of $d_{th} = 1$ nm

$$[O_2^-] = \frac{|\sigma_s|}{F d_{th}}$$

F being Faraday's constant (C $\mathrm{mol^{-1}}$). The concentration of reduced oxygen directly affects the redox potential E_{O_2/O_2^-}, which is updated with the use of the Nernst equation

$$E_F^{OX}(t) = E^0_{O_2/O_2^-} + \frac{RT}{F} \ln \frac{[O_2]}{[O_2^-](t)} \qquad t \in (0,T) \tag{26}$$

$E^0_{O_2/O_2^-}$ being the standard redox potential for the redox couple assuming a constant atmosferic pressure and temperature. $E^0_{O_2/O_2^-} = -0.33$V vs SHE, which corresponds to $E^0 = -0.527$ V vs Ag/AgCl sat for gas oxygen. The concentration of O_2, instead, is supposed to be equal to a constant value corresponding to the measured value of 6.6 $\mathrm{mg\,L^{-1}}$ in oxygenated conditions or equal to 1 $\mathrm{mg\,L^{-1}}$ when fluxing nytrogen in the chamber (see Figure S 2 for more information on

Table S 1: Parameters

**Parameters**	**Units**	**Value**	**Source**
ε	$\mathrm{F\,m^{-1}}$	$3.4\varepsilon_0$	[9]
η_{diss}	–	$3.5 \cdot 10^{-4}$	-
I_0	$\mathrm{mW\,cm^{-2}}$	50	Measured
α	$\mathrm{m^{-1}}$	$1 \cdot 10^{7}$	[10]
V_{OCP}	V vs AgAgCl	0.14	Measured
μ_p	$\mathrm{m^2\,V^{-1}\,s^{-1}}$	$1 \cdot 10^{-8}$	[10]
μ_n	$\mathrm{m^2\,V^{-1}\,s^{-1}}$	$1 \cdot 10^{-12}$	[11]
τ_n, τ_p	s	$1 \cdot 10^{-4}$	[7]
n_i	$\mathrm{m^{-3}}$	$1 \cdot 10^{12}$	Computed
c_{ox}	$\mathrm{mg\,L^{-1}}$	6.6	Measured
λ	eV	0.6	[12]
σ_L, σ_H	eV	0.1	[13]
k_t	$\mathrm{m^4\,s^{-1}}$	$7(\pm 2) \cdot 10^{-30}$	in agreement with [12]
k_p	$\mathrm{m^3\,s^{-1}}$	$3.5(\pm 2.5) \cdot 10^{-23}$	-
m_p^*/m_0	-	1.48	[14]
m_n^*/m_0	-	1.62	[14]

electrode conventions).

3 Crystalline component

The ground-state absorption spectrum of P3HT clorobenzene is shown in Figure S 3, with the continous black line: few earlier works [15, 16] utilizes absorption spectroscopy as a simple probe of how thin-film microstructure varies with processing condition, providing a quantitative estimates not only of the fraction of the film made up of aggregates, but also the degree of excitonic coupling within the aggregates, a parameter which is related to average conjugation length and the crystalline quality. As in Bargigia et al. [17], the high-energy part of the spectrum is attributed to the presence of disordered chains, whereas π-stacked aggregates mainly contribute to the low-energy region. By using the model of weakly interacting H-aggregates developed

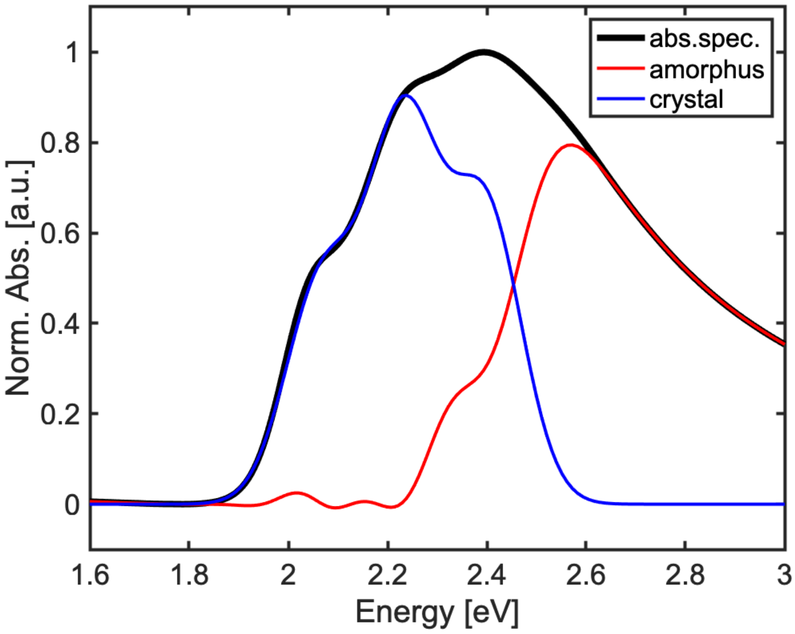

Figure S 3: Absorption spectrum of a film of P3HT of aroung 100 nm in clorobenzene and the estimated component for its crystalline and amorphus part.

by Spano and co-workers [15, 16] we have been able to extract its spectral components and to estimate a percentage of crystalline phase of 41%.

4 Mott-Schottky

In order to characterize the doping levels of our semiconductor, we have performed a Mott-Shottky analysis of a Glass/ITO/P3HT sample 150 nm thick. EIS measurement have been recorded at different fixed potentials, ranging from −0.7 V to +0.7 V vs Ag/AgCl KCl sat. with the potentiostat described in section *Methods*. To perform this analysis, we have utilized a sealed chamber with a hole for the passage of light of 11.2 mm. Before the measurement, the sample has undergone a light soaking of 2 hours at intense light. In Figure S 4, the inverse of the space charge capacitance of P3HT, C, measured in F, is reported against the applied voltage, measured in V vs AgAgCl KCl sat., at three different frequencies. By interpolation of the linear parts of the curves, it is possible to retrieve both the Flat Band potential, namely $E_{fb} = 0.47(\pm 0.05)V$, and the doping concentration, estimated for the 15 KHz curve to be $N_D = 3 \cdot 10^{23}$

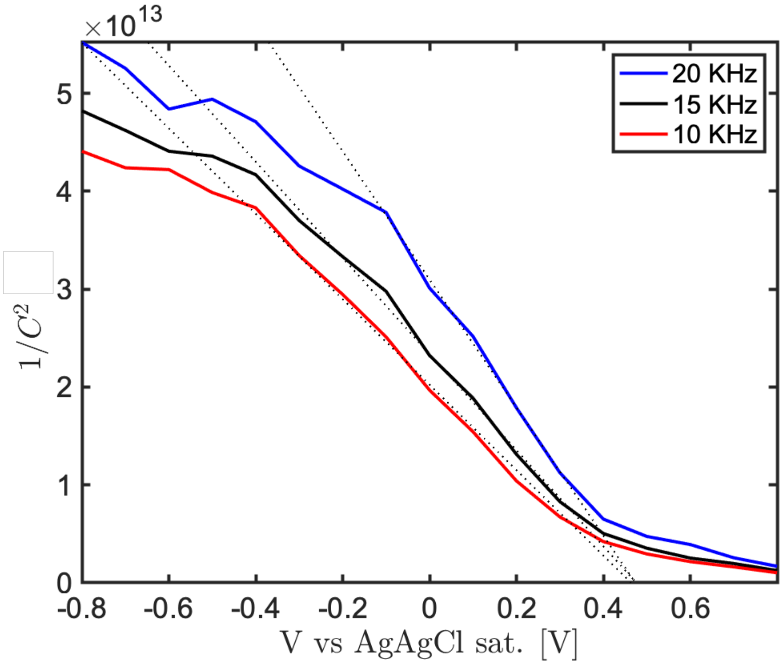

Figure S 4: Mott-Shottky plot of P3HT samples at three different frequency. The applied potential is measured against the AgAgCl KCl sat. electrode and the capacitance is measured in Farad.

m^{-3}, ($\pm 0.4 \cdot 10^{23}$ for the other frequencies). This value is in agreement with what reported in Ghezzi et al. [18]. In Choi et al. [19] the reported values for doping in P3HT are 1 order of magnitude smaller than our: this can be attributed to the fact that our samples have undergone 2 hours of light soaking under a very intense light, thus maximizing the unintentional photodoping of the system. In addition to this, their experiment is undergone in acetonitrile, while our experiment is performed in an aqueous solution: at the interface with the aqueous solution, a reversible trapping fenomena is possibly occurring, thus enhancing the value of our traps concentration.

Geometrical and Interface capacitance The geometrical capacitance has been estimated $\simeq$ 44 nF from:

$$C_G = \frac{\varepsilon \varepsilon_r A}{t} \tag{27}$$

being ε the dielectric constant in vacuum ($\mathrm{F\,m^{-1}}$), ε_r the relative dielectric constant of P3HT reported in Table S 1, A the area of the light-exposed sample ~ 1.9 cm^2, t the thickness of the P3HT layer, equal to 140 nm. From the Mott-Schottky plot for a potential around the OCP value, we can derive $1/C^2 = 1 \cdot 10^{13}$ F^{-2}, namely $C = 316$ nF. With this value from Eq. 27, we can estimate the length of the diffuse interface at the electrolyte around $\sim$ 19 nm.

5 Conductivity Measures

To better understand the role of doping in our systems, we measured their DC conductivity σ_{el} ($\mu\mathrm{S\,cm^{-1}}$) as produced and after exposure to white light ($\sim 1\mathrm{kW\,m^{-2}}$) for 2 hours (to rule out eventual doping during measure). P3HT layers were spin coated over glass and silver paste was added to the side of the sample in order to leave without contact a rectangular surface of each sample. Samples were assumed to be 150 nm thick. Figure S 5 shows the $I-V$ curves acquired using a source measure unit. The voltage has been swept from -1 to $+1$ V, well below P3HT bandgap (between 1.8 and 2 eV). The curves are almost completely linear. Table S 2 reports the slope of the $I-V$ curve, the size of the samples and the calculated conductivities.

By these data we calculated a mean conductivity of 9 ± 2 $\mu\mathrm{S\,cm^{-1}}$ before light exposure and 123 ± 16 $\mu\mathrm{S\,cm^{-1}}$ after a long exposure to very intense light. Since we

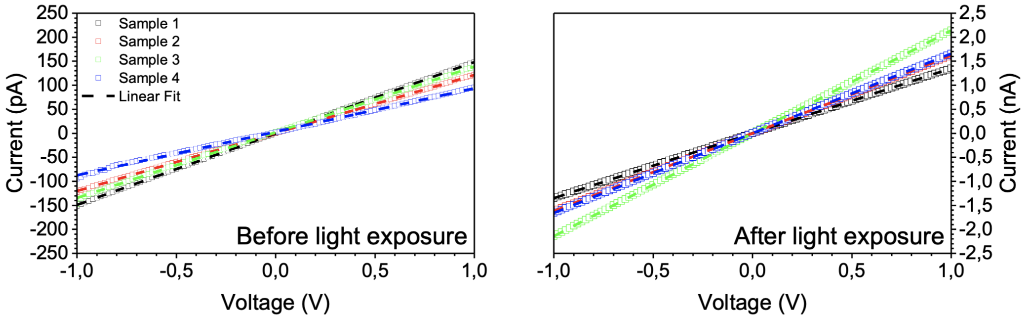

Figure S 5: I-V curves of P3HT samples spin coated over glass and contacted using silver paste.

Table S 2: Parameters of the samples used to measure P3HT conductivity

**Sample**	**Width** (mm)	**Length** (mm)	**Slope before** (nS)	σ_{el} **before** (μS cm^{-1})	**Slope after** (nS)	σ_{el} **after** (μS cm^{-1})
1	17	20	0.15	11.7	1.35	105.9
2	17	20	0.12	9.5	1.62	127.0
3	20	20	0.14	9.1	2.14	142.8
4	19	20	0.09	6.4	1.66	116.4

are probing well below P3HT bandgap, we assume that the conductivity depends only upon doping level. Long exposure to strong light indeed formed long lived charged mobile species but even after a very intense exposure we find that the conductivity we measure is orders of magnitude below the one obtained in literature doping P3HT even at mild levels [20, 21]. Due to this we assumed that unintentional doping has weak effect upon the photophysics of the system.

6 FTIR measures

We conducted a further investigation about photodoping of the system. To check if light exposure can create long lived charged species, we measured ATR-FTIR response of P3HT on glass before and after exposure to 50 mW cm^{-2} of 560 nm light for 3 hours. Figure S 6 reports the result of the analysis. Spectra were acquired using a

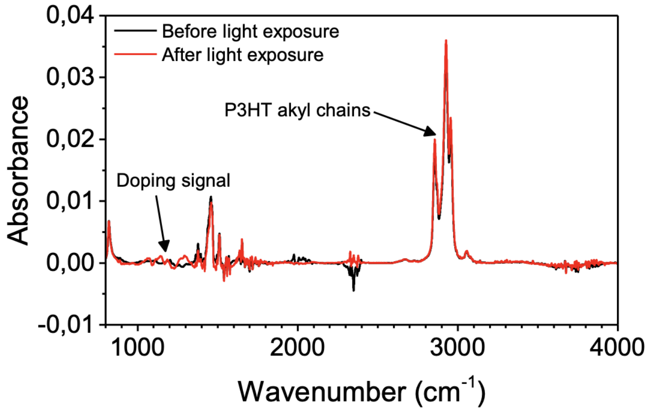

Figure S 6: Absorption spectra of P3HT films before and after light exposure

Bruker Vertex 70 and multiple CRY:Ge crystals for ATR. The signal was acquired fluxing nitrogen inside the optical path and averaged 100 times. FTIR spectra were numerically treated before being reported in this paper. A baseline was calculated fitting a polyline to different points of each spectra and was then subtracted by them. The spectra reveal a strong signal in $2800 - 3100$ cm^{-1} region due to P3HT akyl chains. Interesting for our measures it is the signal that appear after light exposure in the region between $1000 - 1500$ cm^{-1}. Such signal, having a very convoluted shape appears only after light exposure and can be directly linked to the presence of doping induced absorption bands [22]. Such bands are an indication of doping, however, their signal appear to be quite weak even after a long light exposure.

7 Photovoltage with different electrolytes

Figure S 7 compares the PV curves of a P3HT sample immersed in (i) a water based solution with NaCl 0.2 M (ii) a water based solution with Tetrabutylammonium hexafluorophosphate (TBA) 2.5 mM. The exposure and measurement conditions are those reported in the section *Methods* in the article. A proposed mechanism of recovery of electroneutrality in organic polymers in electrolyte is the anion (or cation) intercalation. In order to test whether this can be an effective mechanism in our device we have performed the PV measurement with an electrolyte with high steric hindrance, namely TBA 2.5 mM. Due to the huge volume occupied by these salts, no intercalation

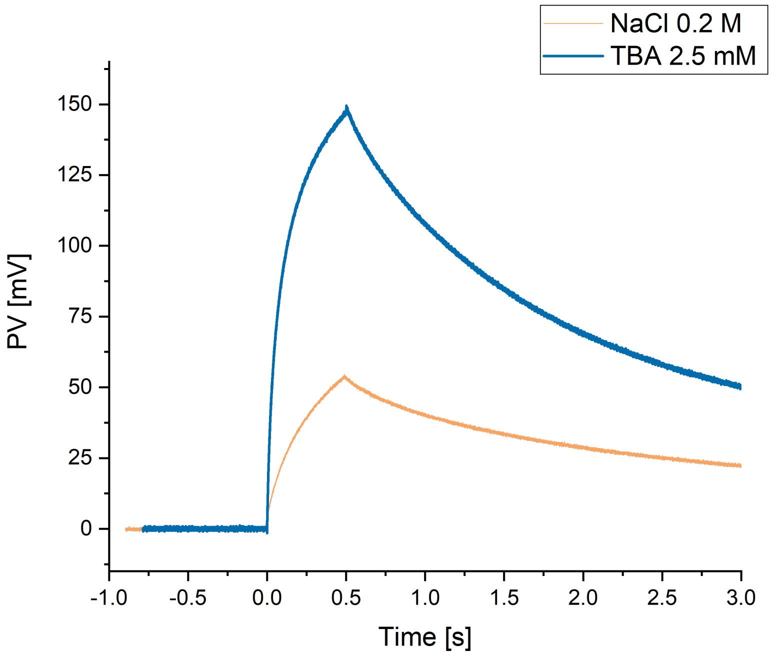

Figure S 7: Photovoltage measurement of a P3HT sample in water (i) NaCl 0.2 M; (ii) TBA 2.5 mM.

is supposed to occur. But looking at the blue curve one observes a good recovery of the PV: intercalation of anions is hindered with TBA, therefore a second mechanism should be taken into account. Surface recombination of holes is therefore accounted as the mechanism responsible for electroneutrality recovery and the mechanism is better described in Sec. 2.

8 Additional simulation results

Figure S 8 shows the drift and diffusion component of the estimated $\mathbf{J}_p$ after 1 and 5 µs of illumination. Results show that in the timescale of µs the diffusion component is predominant, being 3 orders of magnitude larger than the drift component, thus ensuring a redistribution of holes across the bulk immediately after illumination. Figure S 9 instead shows how, in the ms timescale, the two components are equal and opposite, thus leading to an almost zero internal flux of holes. When diffusion and drift currents start balance each other, we observe that holes tend to accumulate near the electrolyte

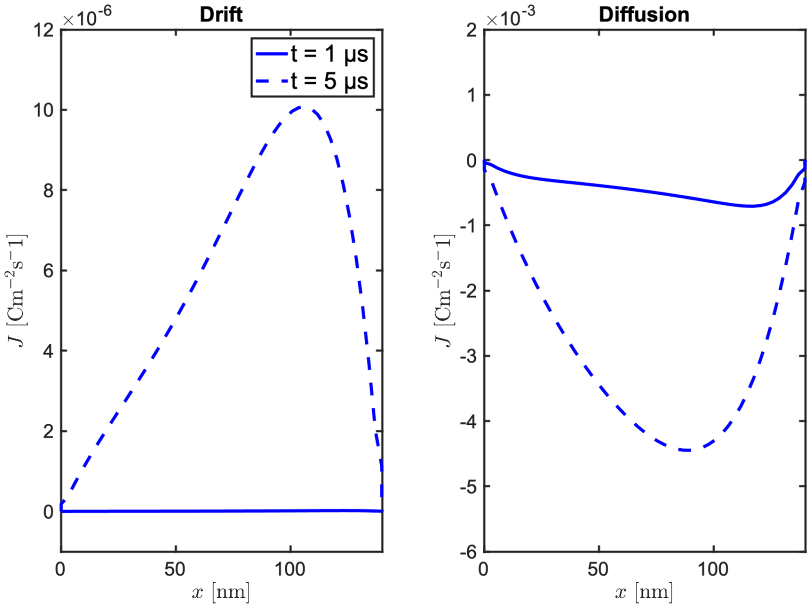

Figure S 8: The figure reports the simulation of the drift and diffusion component of the hole current density after 2 and 5 μs of illumination for a sample of 140 nm illuminated by the electrolyte side.

interface. Here due to the effects of the boundary conditions, we also observe the electric field increasing (see Figure S 10).

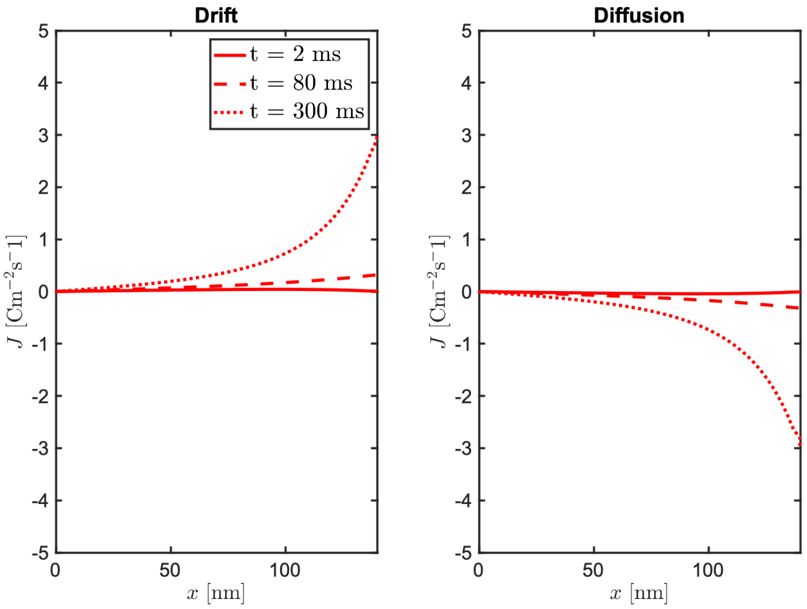

Figure S 9: The figure reports the simulation of the drift and diffusion component of the hole current density after 2, 80 and 300 ms of illumination for a sample of 140 nm illuminated by the electrolyte side.

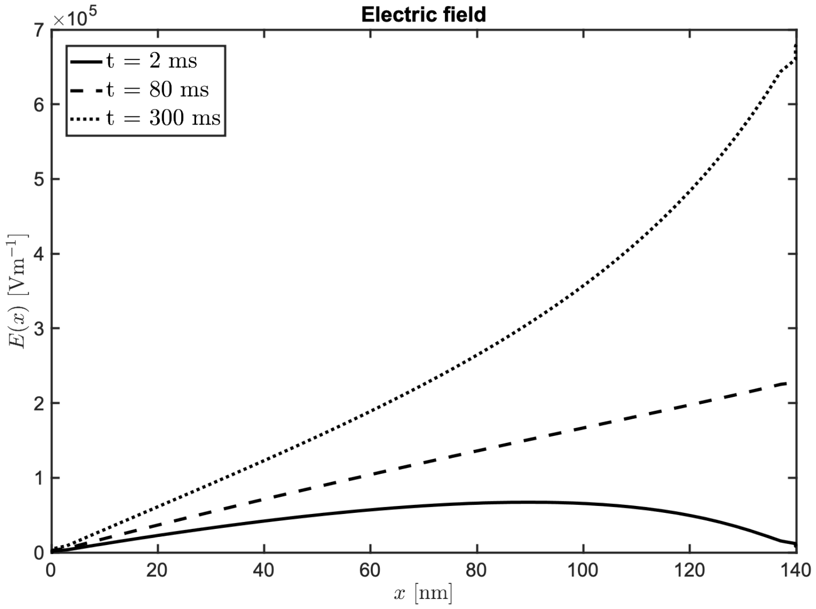

Figure S 10: The figure reports the simulation of the electric field after 2, 80 and 300 ms of illumination for a sample of 140 nm illuminated by the electrolyte side.

References

(1) Selberherr, S., *Analysis and Simulation of Semiconductor Devices*; 9; Springer-Verlag: Wien New York, 1984; Vol. 53, pp 1689–1699, arXiv: `arXiv:1011.1669v3`.

(2) Markowich, P. A., *The Stationary Semiconductor Device Equations*; Springer-Verlag: 1986.

(3) Markowich, P. A.; Ringhofer, C. A.; Schmeiser, C., *Semiconductor Equations*, 1st ed.; Springer-Verlag Wien: 1990.

(4) Jerome, J. W., *Analysis of Charge Transport*; Springer: 1996.

(5) Braun, C. L. *The Journal of Chemical Physics* **1984**, *80*, 4157–4161.

(6) Hwang, I.; Greenham, N. C. *Nanotechnology* **2008**, *19*, DOI: `10.1088/0957-4484/19/42/424012`.

(7) MacKenzie, R. C.; Shuttle, C. G.; Chabinyc, M. L.; Nelson, J. *Advanced Energy Materials* **2012**, *2*, 662–669.

(8) Memming, R., *Semiconductor Electrochemistry*; VCH, W., Ed., 2001.

(9) Schneider, M.; Wagenpfahl, A.; Deibel, C.; Dyakonov, V.; Schöll, A.; Reinert, F. *Organic Electronics* **2014**, *15*, 1552–1556, `http://dx.doi.org/10.1016/j.orgel.2014.03.012`.

(10) Popescu, B. V.; Popescu, D. H.; Lugli, P.; Locci, S.; Arca, F.; Tedde, S. F.; Sramek, M.; Hayden, O. *IEEE Transactions on Electron Devices* **2013**, *60*, 1975–1981.

(11) Agostinelli, T.; Caironi, M.; Natali, D.; Sampietro, M.; Biagioni, P.; Finazzi, M.; Duò, L. *Journal of Applied Physics* **2007**, *101*, DOI: `10.1063/1.2738429`.

(12) Rudolph, M.; Ratcliff, E. L. *Nature Communications*, DOI: `10.1038/s41467-017-01264-2`, `http://dx.doi.org/10.1038/s41467-017-01264-2`.

(13) Blakesley, J. C.; Greenham, N. C. *Journal of Applied Physics* **2009**, *106*, 1–8.

(14) Xie, X.-h.; Shen, W.; He, R.-x.; Li, M. **2015**, DOI: `10.5012/bkcs.2013.34.10.2995`.

(15) Clark, J.; Silva, C.; Friend, R. H.; Spano, F. C. *Physical Review Letters* **2007**, *98*, 1–4, arXiv: `0702663 [cond-mat]`.

(16) Clark, J.; Chang, J. F.; Spano, F. C.; Friend, R. H.; Silva, C. *Applied Physics Letters* **2009**, *94*, 2007–2010, arXiv: `0903.1670`.

(17) Bargigia, I.; Zucchetti, E.; Kandada, A. R. S.; Moreira, M.; Bossio, C.; Wong, W. P.; Miranda, P. B.; Decuzzi, P.; Soci, C.; D'Andrea, C.; Lanzani, G. *ChemBioChem* **2019**, *20*, 532–536.

(18) Ghezzi, D.; Antognazza, M. R.; Maccarone, R.; Bellani, S.; Lanzarini, E.; Martino, N.; Mete, M.; Pertile, G.; Bisti, S.; Lanzani, G.; Benfenati, F. *Nat Photonics* **2013**, *7*, 400–406.

(19) Choi, W. T.; Bard, A. J. *The Journal of Physical Chemistry C* **2020**, *124*, 3439–3447.

(20) Lim, E.; Peterson, K. A.; Su, G. M.; Chabinyc, M. L. *Chemistry of Materials* **2018**, *30*, 998–1010.

(21) Hynynen, J.; Kiefer, D.; Yu, L.; Kroon, R.; Munir, R.; Amassian, A.; Kemerink, M.; Müller, C. *Macromolecules* **2017**, *50*, 8140–8148.

(22) Enengl, C.; Enengl, S.; Pluczyk, S.; Havlicek, M.; Lapkowski, M.; Neugebauer, H.; Ehrenfreund, E. *ChemPhysChem* **2016**.
